# Thyroid‐Targeted Nano‐Bombs Empower HIFU for Graves' Disease

**DOI:** 10.1002/advs.202414597

**Published:** 2025-01-22

**Authors:** Binhao Wang, Zhuobing Yin, Xiangyue You, Hanwei Peng, Ying Jiang

**Affiliations:** ^1^ The Department of Head and Neck Surgery Cancer Hospital of Shantou University Medical College Shantou Guangdong 515041 P. R. China

**Keywords:** graves' disease, HIFU, nano‐bomb, thyroid‐targeted

## Abstract

Graves' disease (GD) is an autoimmune disorder with a high incidence rate, particularly affecting women of reproductive age. Current treatment modalities for GD carry significant disadvantages, especially for pregnant or nursing women. As a novel extracorporeal therapeutic technique, high‐intensity focused ultrasound (HIFU) shows great promise for treating GD; however, its low treatment efficacy impedes clinical application. In this study, a thyroid‐targeted nano‐bomb system (PD‐PLGA@Si‐Ab/PDA‐I, PSAPI) is developed to enhance HIFU efficacy and improve therapeutic outcomes for GD. The core structure of PSAPI encapsulates a phase‐transition material, perfluorohexane, and the anti‐inflammatory drug diclofenac within a poly(lactide‐co‐glycolide) (PLGA) and silica shell. A polydopamine coating enhances biocompatibility, while iodine loading and thyroid‐stimulating hormone receptor (TSHR) antibodies grafting ensure targeted delivery to the thyroid. Robust in vitro and in vivo results demonstrated that PSAPI is highly biocompatible, accumulates in the thyroid within 24 h after administration, and significantly potentiates the therapeutic efficacy of HIFU, resulting in markedly reduced inflammatory responses. Transcriptomic analysis revealed a cellular defense mechanism activated in PSAPI‐treated cells following HIFU irradiation, highlighting potential molecular targets for the future development of HIFU‐sensitizing agents. The biocompatible PSAPI nano‐bomb developed in this study holds great transformative potential, addressing critical gaps in current therapeutic practices for GD.

## Graphical abstract

To promote the application of HIFU on GD therapy, this study develops a thyroid‐targeted nano‐bomb system (PSAPI) with a core of PFH and DC, encapsulated in a PLGA‐silica shell, further coated with PDA and modified with iodine and TSHR antibodies. In vitro and in vivo results show PSAPI's excellent stability, biocompatibility, and functionality, making it a promising adjuvant for HIFU in the treatment of GD.

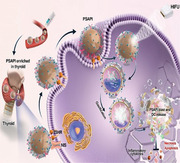

## 1 Introduction

Graves' disease (GD) is an autoimmune disorder caused by the stimulation of autoantibodies against the thyroid‐stimulating hormone (TSH) receptor on thyroid follicular cells, leading to excessive thyroid hormone production (hyperthyroidism).^[^ ^1^ ^]^ GD is the most common cause of hyperthyroidism, affecting over 1% of the population, with an incidence ranging from 20–50 per 100 000 per year.^[^ ^2^, ^3^ ^]^ It occurs more commonly in women than in men by a ratio of 6–7 to 1, particularly in women of reproductive age between 20–50 years.^[^ ^4^ ^]^ Established treatment modalities for GD include antithyroid drugs (ATDs), which block thyroid hormone synthesis and release; radioactive iodine (RAI); and total or subtotal thyroidectomy, which destroy or remove the thyroid.^[^ ^5^, ^6^ ^]^ When considering the preferred initial treatment for GD, ATDs primarily target symptom control, and relapses are common, occurring in approximately 30–40% of cases within the first 12 months and in 50–60% of cases in the longer term.^[^ ^7^, ^8^ ^]^ RAI evokes fear of radiation and is not suitable for pregnant or nursing patients,^[^ ^9^ ^]^ as well as those with severe eye disease.^[^ ^10^, ^11^ ^]^ Thyroidectomy often results in hypothyroidism, necessitating lifelong thyroid hormone replacement and clinical monitoring.^[^ ^12^, ^13^ ^]^ Patient dissatisfaction and reduced quality of life urge the development of therapeutic advances for GD.

As a novel extracorporeal therapeutic technique, high‐intensity focused ultrasound (HIFU) holds great promise for treating GD. The underlying mechanism of HIFU is to create a high‐intensity ultrasound focus within the body using external transducers, thereby inducing localized coagulative necrosis with minimal damage to surrounding tissues.^[^ ^14^ ^]^ HIFU is a non‐invasive, non‐ionizing ablation technique with deep tissue penetration and low side effects, making it a safer option for pregnant or nursing patients, as well as for those reluctant to undergo surgery.^[^ ^15^ ^]^ Clinically, HIFU has been used to treat a variety of solid tumors in the breast, liver, kidney, prostate, bone, and uterine fibroids, as well as nervous system disorders.^[^ ^16^, ^17^, ^18^, ^19^, ^20^, ^21^, ^22^ ^]^ The first clinical trial of HIFU's application in thyroid diseases was conducted on benign thyroid nodules by Esnault et al. in 2011.^[^ ^23^ ^]^ Subsequent studies showed that HIFU ablation caused thyroid nodule destruction through irreversible tissue necrosis within the targeted area.^[^ ^24^ ^]^ Inspired by the treatment of benign thyroid nodules, Lang et al. employed HIFU to treat GD and disease remission was observed after a single session of HIFU treatment.^[^ ^25^, ^26^ ^]^ Moreover, unlike RAI and thyroidectomy, none of the patients developed hypothyroidism, indicating the feasibility of applying HIFU as a GD therapy. Unfortunately, the relapse rate was as high as 41.3%, significantly higher than with RAI or thyroidectomy. Therefore, to promote its clinical application in the treatment of GD, HIFU's efficacy needs improvement. Although increasing the acoustic power or exposure time could improve ablation efficacy, it also elevates the risk of complications, including pain, neck discomfort, vocal cord palsy, skin redness, and swelling.

Engineered nanoparticles (NPs) have been widely studied and used to facilitate disease diagnosis and treatment. A variety of NPs have been shown to enhance ultrasound responsiveness, and therefore, boost the efficiency of HIFU ablation.^[^ ^27^ ^]^ One of critical underlying mechanisms behind the HIFU nano‐enhancers is to amplify the thermal, mechanical, and cavitational effects by introducing bubble‐generating materials.^[^ ^28^ ^]^ Inspired by this, our study presents a novel organic‐inorganic hybrid nanosystem designed to advance the therapeutic application of HIFU in GD therapy. The core of this system is perfluorocarbon (perfluorohexane, PFH), which undergoes a liquid‐to‐gas phase transition triggered by ultrasound‐induced heat. PFH is encapsulated by a poly(lactide‐co‐glycolide) (PLGA) and silica shell, combining the high cargo retention and biocompatibility of PLGA with the mechanical strength of silica. Triggered by ultrasound, the expanding PFH gas creates pressure that accumulates within the rigid silica shell, eventually reaches the breaking point and causes the shell to rupture abruptly, mimicking a bomb blasting on a nanoscale. The instant blast overpressure intensifies the thermal and mechanical effects of HIFU while the liberated gas bubble amplifies cavitation effect.

Inflammatory responses induced by HIFU ablation are common and can be severe, potentially life‐threatening in thyroid‐related diseases due to airway obstruction caused by swelling. Hence, diclofenac (DC), a potent anti‐inflammatory agent, was co‐encapsulated with PFH (PD‐PLGA@Si, PS), designed to mitigate localized inflammation post‐ablation. Our ultrasound‐triggered nano‐bomb system enables precise, controlled release of DC, increasing its localized concentration at the ablation site. This targeted release mechanism effectively suppresses in situ inflammatory responses, enhancing not only the therapeutic efficacy but also the safety of HIFU ablation. The surface of the nano‐bomb was modified with polydopamine (PDA) to improve biocompatibility and facilitate the attachment of iodide ions, thereby imparting thyroid‐targeting capability to the NPs. Additionally, thyroid‐stimulating hormone receptor (TSHR) monoclonal antibodies were grafted onto the silica shell, further enhancing the specificity and precision of thyroid targeting. Consequently, the thyroid‐targeted nano‐bomb system (PD‐PLGA@Si‐Ab/PDA‐I, PSAPI) developed in this study represents a promising enhancer for HIFU, thereby advancing the clinical application of HIFU in the treatment of GD.

## 2 Results and Discussion

### 2.1 Synthesis and Characterization of PSAPI

**Scheme 1:**
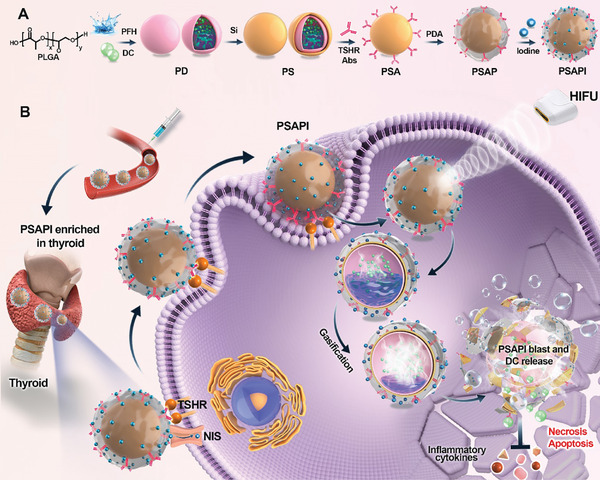
Schematic illustration of the synthesis process and therapeutic mechanism of PSAPI.

**Figure 1:**
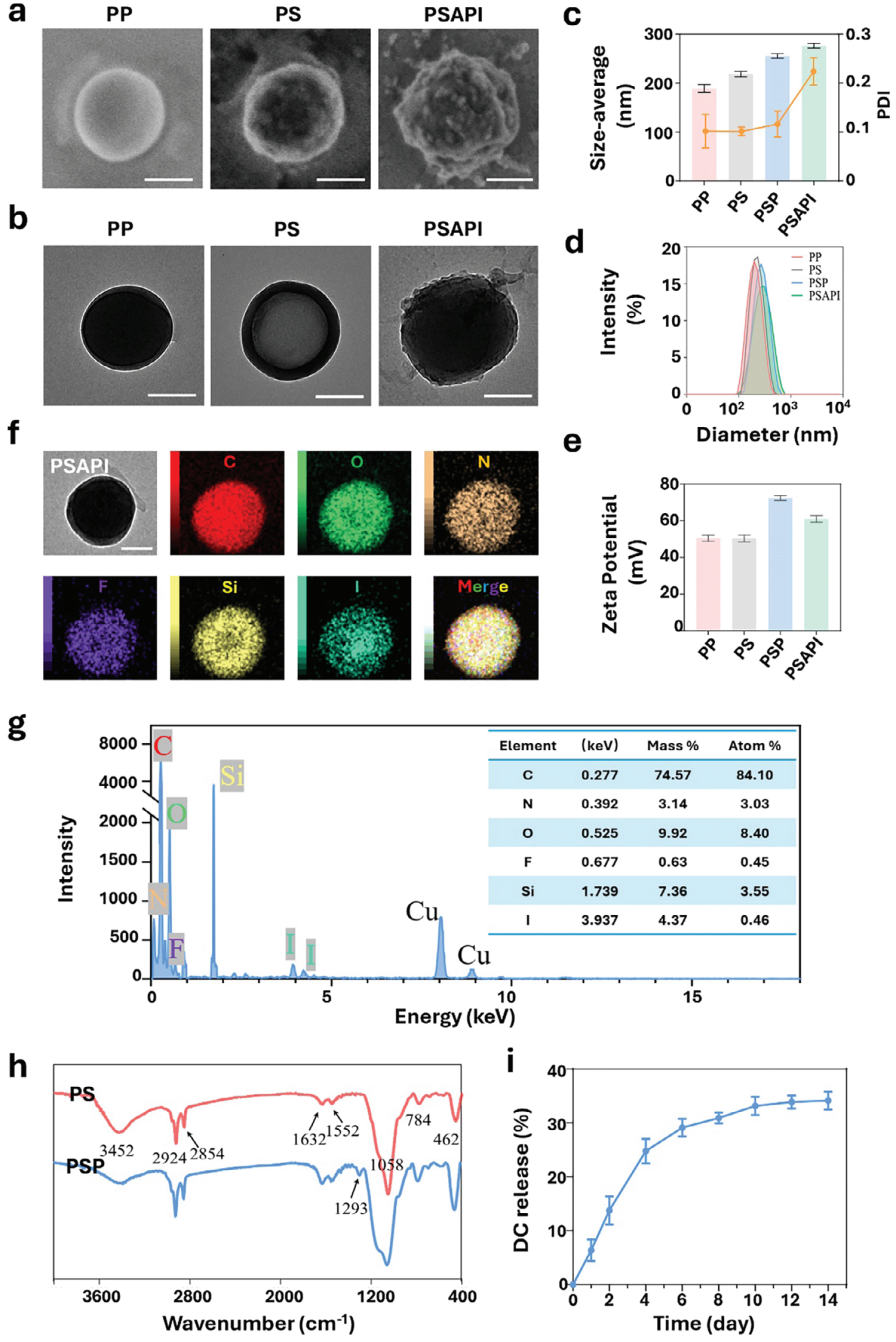
Characterization of PSAPI. a) SEM and b) TEM images of NPs. c) Average size and polydispersity index (PDI), d) dynamic light scattering (DLS) profile, and e) zeta potential of NPs. f) EDS element mapping images and g) EDS Point scan spectrum and elemental analysis of PSAPI. h) FTIR spectra of PS and PSP. i) DC release curve in PBS over a 14‐day period. Scale bar: 100 nm.

The synthetic scheme of PSAPI is illustrated in **Scheme** **1**. First, PD‐PLGA (PP) nanospheres were produced using the oil‐in‐water (O/W) single emulsion solvent evaporation method. PFH encapsulation was confirmed by the presence of the fluoride element in energy dispersive X‐ray spectroscopy (EDS) elemental mapping imaging (Figure S1a, Supporting Information) and EDS elemental point analysis (Figure S1b, Supporting Information). The encapsulation efficiency of DC was ≈50%, as shown in Figure S2 (Supporting Information). The release of encapsulated DC in PBS over a 14‐day period was ≈34% (**Figure** **1**i). The silica shell was formed by the hydrolysis and co‐condensation of tetraethyl orthosilicate (TEOS) and (3‐aminopropyl)triethoxysilane (APTES),^[^ ^29^ ^]^ which introduces amino groups for antibody coupling. Under transmission electron microscopy (TEM), a thick silica shell was visualized. EDS elemental point analysis revealed the presence of Si element on PD‐PLGA@Si (PS) (Figure S3, Supporting Information). As shown in Figure 1h, the fourier transform infrared spectroscopy (FTIR) spectra of PS showed characteristic absorption bands for Si─O stretching and bending vibrations at 784 and 462 cm^−1^.^[^ ^30^ ^]^ Intense band ≈1058 cm^−1^ was attributed to stretching vibrations of Si─O─Si bridges.^[^ ^31^, ^32^ ^]^ The N─H bending vibrations of amine groups were shown at 1552 cm^−1^.^[^ ^33^, ^34^ ^]^ The peak at 1632 was due to O─H stretching of surface silanol groups with hydrogen bond or the remaining adsorbed water molecules.^[^ ^35^ ^]^ Absorbance at 3452 cm^−1^ was associated with N─H stretching of amine groups or O─H stretching.^[^ ^36^ ^]^ Asymmetrical and symmetrical stretching vibrations of CH_2_ groups were shown at 2924 and 2854 cm^−1^ respectively.^[^ ^37^ ^]^ Dopamine undergoes oxidative polymerization in an alkaline environment and forms an adhesive PDA coating on top of PS (PD‐PLGA@Si/PDA, PSP). The FTIR spectra of PSP display characteristic PDA signals of phenolic C‐OH stretching at the peak of 1293 cm^−1^,^[^ ^38^ ^]^ confirming the successful modification of the surface with PDA (Figure 1h).

TSHR antibodies were grafted onto the silicon shell (PD‐PLGA@Si‐Ab, PSA) via amine coupling method with 1‐Ethyl‐3‐(3‐dimethylaminopropyl)carbodiimide hydrochloride/N‐hydroxysuccinimide (EDC/NHS) reagents. Briefly, the carboxyl groups on the antibodies were activated by the EDC/NHS mixture for 15 min. Then, PS was added to the activated antibodies and reacted for 2 h. To confirm successful antibody conjugation, fluorescent secondary antibodies were mixed with PSA to allow binding to the TSHR antibodies. PSA was then visualized under microscope. Compared to PS, fluorescent signals were detected in PSA, indicating the presence of antibodies on the surface of the NPs (Figure S4, Supporting Information). Iodine was loaded onto the PDA coating (PD‐PLGA@Si‐Ab/PDA‐I, PSAPI) through electrostatic interactions between iodine anion and the positively charged lysine and histidine residues, which are rich on PDA.^[^ ^39^, ^40^ ^]^ EDS elemental point analysis verified the presence of iodine on PSAPI (Figure 1f,g).

The color changes of the NPs were observable to the naked eye. The initial PP dispersion appeared white, transitioning to a light yellow upon the introduction of the silica shell. Subsequent modification with PDA resulted in a further color shift to dark brown. Notably, iodine loading and antibody grafting did not induce any discernible change in the color of the NPs (Figure S5a, Supporting Information). The morphology of the NPs was visualized by scanning electron microscope (SEM) and TEM. PP nanospheres exhibited smooth surfaces with an average diameter of ≈190 nm (Figure 1c). TEM images of PS revealed a silica shell encapsulating the PP nanospheres, which led to a rougher surface in SEM images. PSAPI demonstrated even greater surface roughness due to the PDA coating (Figure 1a,b). The particle sizes of PS, PSP, and PSAPI showed a slight increase but remained within the range of 200–300 nm. The polydispersity index (PDI) of PP, PS, and PSP remained ≈0.1, indicating good size uniformity (Figure 1c,d). However, the PDI of PSAPI increased to above 0.2 due to the loading of iodine ions, which not only introduced anions but also compressed the electrical double layer (EDL),^[^ ^41^ ^]^ resulting in a decrease in zeta potential (Figure 1e) and reduced electrostatic repulsion, thereby leading to increased aggregation. Over a 4‐week period, no significant change in particle dimensions was observed, demonstrating good stability of the NPs (Figure S5b, Supporting Information).

### 2.2 Ultrasound Induced PSAPI Explosion

**Figure 2:**
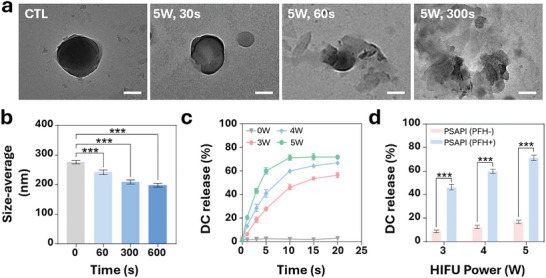
HIFU triggered PSAPI collapse and DC release. a) Representative TEM images of PSAPI under HIFU irradiation (5 W) for 30, 60, and 300 s. b) Average size of PSAPI before and after HIFU irradiation (5 W). c) DC release curves from PSAPI under HIFU irradiation at the 0, 3, 4, and 5 W for various time points. d) DC release from PFH negative and positive PSAPI under irradiation with different HIFU power for 10 min. Data are presented as mean ± SD (n ≥ 3). Statistical significance was determined by one‐way ANOVA followed by Dunnett's multiple comparison test or two‐way ANOVA followed by Bonferroni multiple comparison test. ns, not significant, ^***^ *p* < 0.001. Scale bar: 100 µm.

Ultrasound increases in situ tissue temperature, inducing the gasification of PFH. The gasified PFH raises the internal pressure of PSAPI, ultimately rupturing the silica shell, exploding, and releasing gas bubbles. To study this, we first evaluated the bubble release from PSAPI under thermal conditions. Polyacrylamide gel incorporated with PSAPI was heated to 60 °C for 300 s and observed under a microscope. As shown in Figure S6 (Supporting Information), gas bubbles emerged post‐heating, indicating that the encapsulated PFH in PSAPI underwent a liquid‐to‐gas phase transition. We then examined the morphological changes in PSAPI after HIFU irradiation using TEM. With increasing radiation power and exposure time, PSAPI lost its spherical morphology, ruptured, and fragmented into smaller pieces and polymeric clouds (**Figure** **2**a). The particle size decreased accordingly (Figure 2b). Upon PSAPI collapse, the release of DC was investigated. Under HIFU irradiation (5 W, 5 min), >60% of the encapsulated DC was released, implying the successful destruction of PSAPI (Figure 2c). On the contrary, PSAPI without encapsulated PFH released minimal amounts of DC under the same HIFU power and duration (Figure 2d), showing that the presence of encapsulated PFH is critical for PSAPI explosive rupture.

### 2.3 In Vitro Cytotoxicity

**Figure 3:**
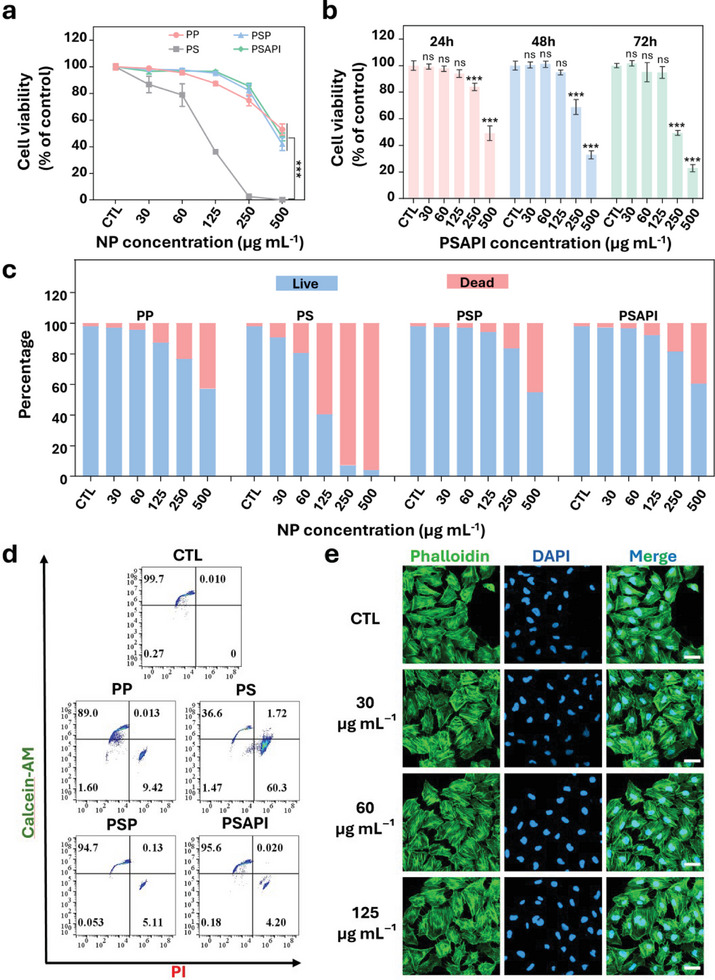
In vitro cytotoxicity analysis. a) Relative cell viability following incubation with NPs for 24 h. b) Relative cell viability following incubation with PSAPI at various concentration for 24, 48, and 72 h. c) Relative live/dead cell percentage following incubation with different concentrations of NPs for 24 h. d) Flow cytometric analysis of cells after incubation with NPs at the concentration of 125 µg mL⁻¹ for 24 h. e) Morphology of cells after PSAPI incubation. Data are presented as mean ± SD (n ≥ 3). Statistical significance was determined using one‐way ANOVA followed by Dunnett's multiple comparison test or two‐way ANOVA followed by Bonferroni multiple comparison test. ns, not significant, and ^***^ *p* < 0.001 versus CTL (b). Scale bar: 40 µm.

To assess the biocompatibility of NPs, we incubated NPs with human thyroid follicular epithelial cell line Nthy‐ ori 3‐1 for 24 h at varying concentrations, followed by measurement of cell viability using the cell counting kit‐8 (CCK‐8) assay. As shown in **Figure** **3**a, the introduction of a silica shell (PS) significantly inhibited cell viability (>60% reduction) at a concentration as low as 125 µg mL^−1^. In contrast, surface modification with PDA effectively reversed this inhibitory effect, resulting in enhanced biocompatibility of the NPs. When incubated with PDA‐modified NPs (PSP, PSAPI) at concentrations up to 250 µg mL^−1^, cell viability still exceeded 80%. Additionally, a time‐course study was performed on PSAPI to investigate the impact of extended exposure. No significant changes in cell viability were observed over a 72‐h incubation period at concentrations below 125 µg mL^−1^ (Figure 3b). As the CCK‐8 assay quantifies viable cell numbers, which can be confounded by variations in proliferation rates, it may not fully capture NP‐induced cytotoxicity. To further validate these findings, we conducted live/dead assays using calcein‐AM and propidium iodide (PI) staining. Quantification of fluorescent signals via plate reader revealed a significant increase in dead cells in the PS treatment group, whereas in the PDA‐modified NP groups (PSP, PSAPI), the percentage of dead cells remained below 5% at concentrations ≤125 µg mL^−1^ (Figure 3c). These results were corroborated by flow cytometry (Figure 3d) and fluorescence microscopy (Figure S7, Supporting Information). The morphology of PSAPI incubated cells were visualized by phalloidin staining. Compared to control, the shape of cells treated with PSAPI were intact. F‐actin stress fiber of all groups remained distinct. No obvious morphologic alteration was observed (Figure 3e). The CCK‐8 assay was also extended to THP‐1 (human acute monocytic leukemia cell line), HEK‐293T (human embryonic kidney cell line), and HUVEC (human umbilical vein endothelial cells). Consistent with the results observed in thyroid cells, no significant reduction in cell viability was detected when PSAPI was administered at concentrations below 125 µg mL^−1^(Figure S8, Supporting Information).

In conclusion, PDA surface modification substantially improves the biocompatibility of NPs, with PSAPI exhibiting minimal cytotoxicity at concentrations ≤125 µg mL^−1^. Consequently, all subsequent experiments were performed using NPs concentrations of 125 µg mL^−1^.

### 2.4 Thyroid Targeting

To enhance the therapeutic efficacy of HIFU in GD, it is crucial to enrich nano‐enhancers within the thyroid gland. Thus, the thyroid‐targeting capability of PSAPI is of paramount importance. PSAPI employs two primary targeting strategies: 1) TSHR antibodies, which specifically bind to TSH receptors on the surface of thyroid follicular cells, and 2) iodine, which is actively transported into thyroid follicular cells via the NIS (Na(+)/I(‐) symporter), a basolateral membrane glycoprotein. The rationale for utilizing dual targeting lies in the fact that two distinct mechanisms for cellular uptake enhance the likelihood of nanoparticle (NP) accumulation within the thyroid gland. Iodide ions at the outer layer of PSAPI are weakly associated with the PDA coating through electrostatic interactions, allowing their facile detachment. Upon encountering the NIS, iodide ions preferentially bind to the symporter due to its high affinity for iodine, driving their active transport into the cell. Consequently, the iodide ions dissociate from the PSAPI surface, thereby no stable interaction between PSAPI and the cell membrane can be formed via iodide‐NIS binding. Instead, the interaction between NIS and iodine hauls NPs to cell surface enhancing the odds of NP internalization. As iodide ions are released from NPs, TSHR antibodies on the PSAPI surface are brought into close proximity to TSHR, promoting specific binding. Given that the TSHR antibodies are covalently conjugated to the silica shell of PSAPI, this enables stable attachment of the PSAPI to the cell surface which largely enhances internalization.

In addition, although TSHR and NIS are highly expressed in thyroid, both are also present in extra‐thyroid tissues. While NIS is predominantly expressed in the thyroid, it is also found in limited tissues such as the lacrimal and salivary glands, lactating breast tissue, and breast cancer cells.^[^ ^42^ ^]^ TSHR is expressed in various extra‐thyroid tissues, including the central nervous system, skin, kidney, liver, immune cells, and adipose tissue.^[^ ^43^ ^]^ To ensure specificity in our nanosystem, we utilized both targets.

To evaluate the thyroid‐targeting specificity and efficiency of PSAPI, the NPs were labeled with Cy2, Cy5, or Cy7 fluorescent dyes, and their cellular internalization was compared between thyroid (Nthy‐ori 3‐1) and non‐thyroid cells (NCM460&BT‐549). NCM460, a normal human colon mucosal epithelial cell line, was chosen due to their lack of TSHR and NIS expression. BT‐549, a breast cancer cell line, expresses TSHR but not NIS was also included as a control (Figure S9, Supporting Information).

**Figure 4:**
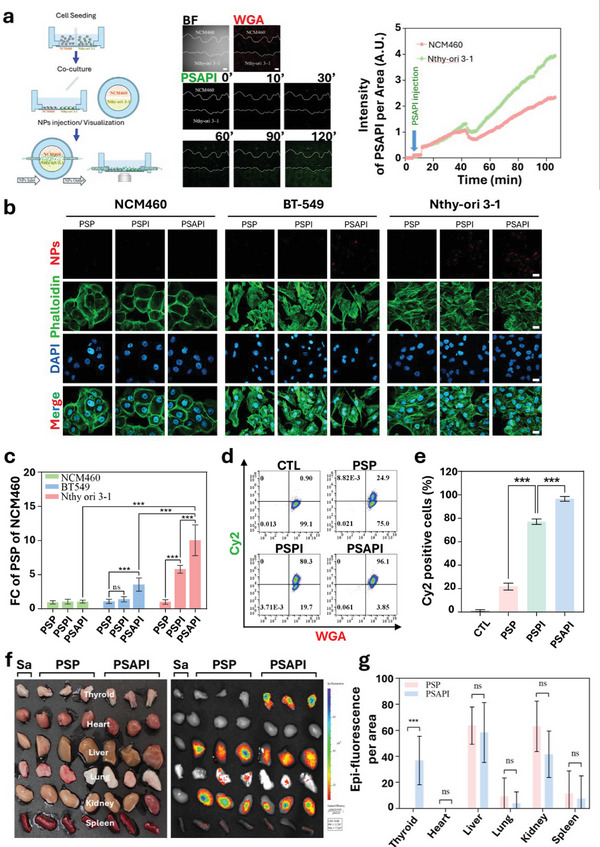
Evaluation of thyroid‐targeting capability of PSAPI. a) Live cell uptake of Cy2‐doped PSAPI between Nthy‐ori 3‐1 and MCN 460 cells. b) Representative confocal microscopy images of MCN460, BT‐549 and Nthy‐ori 3‐1 cells incubated with Cy5‐doped NPs at the concentration of 125 µg mL^−1^ for 24 h. c) Quantitative analysis of internalized Cy5 fluorescence intensity per cell normalized to PSP of NCM460. d) Flow cytometric analysis and e) quantitative analysis of Cy2 positive Nthy‐ori 3‐1 cells. f) Images and g) quantitative epi‐flurescence analysis of major organs (including heart, liver, spleen, lungs, and kidneys) and thyroid after tail vein injection of saline and Cy7‐doped NPs (4 mg kg⁻¹) for 24 h. Data are presented as mean ± SD (n ≥ 3). Statistical significance was determined using one‐way ANOVA followed by Dunnett's multiple comparison test or two‐way ANOVA followed by Bonferroni multiple comparison test. ns, not significant and ^***^ *p* < 0.001. Scale bar: a) 100 µm and b) 20 µm.

To simulate NP uptake in the human bloodstream, we developed a NP pump injection system in which NCM460 and Nthy‐ori 3‐1 cells were co‐cultured on a glass‐bottom dish. A continuous flow of PSAPI (100 µL min⁻¹) was introduced, enabling real‐time visualization of NP uptake via live cell microscopy. In this setup, both thyroid (Nthy‐ori 3‐1) and non‐thyroid (NCM460) cells were exposed to identical conditions, eliminating potential systematic errors associated with separate experiments. The fluid flow rate was determined based on parameters outlined in the methods section. Following PSAPI administration, fluorescent signal accumulation was observed in both cell types. After 30–40 min, the fluorescent intensity in Nthy‐ori 3‐1 cells exceeded that of NCM460 cells. Over a 120‐min period, the fluorescent signal in Nthy‐ori 3‐1 cells reached 1.8 times that in NCM460 cells, indicating preferential uptake of PSAPI by thyroid cells (Video S1, Supporting Information; **Figure** **4**a).

While the pump injection system on live cells displays real‐time NP uptake, the limitation of this assay is the challenge in distinguishing between fluorescent signals from internalized and non‐internalized NPs. To address this, we fixed the cells and performed a washout step to remove un‐internalized NPs. Signals inside Nthy‐ori 3‐1 (TSHR+/NIS+), BT‐549 (TSHR+/NIS‐) and NCM460 (TSHR‐/NIS‐) were measured. Compared to PSP, PSPI (TSHR antibody‐/I+) significantly increased fluorescent signal intensity in Nthy‐ori 3‐1 cells, while no appreciable increase was observed in NCM460 and BT‐549 cells revealing the specific interaction of NIS and iodine. The NP internalization was further enhanced by introducing TSHR antibodies (PSAPI) in Nthy‐ori 3‐1 cells but not NCM460 cells which suggest that the association of TSHR and its antibody facilitates NP internalization. This is consistent with the increased signal in BT‐549 cells (Figure 4b,c). Flow cytometry results further demonstrated that only 22% of cells internalized PSP. In contrast, iodine loading alone or with TSHR antibody conjugation drastically enhanced NP uptake, with 78.8% and 96.6% of cells exhibiting NP internalization, respectively. These findings suggest that both iodine incorporation and TSHR antibody grafting substantially improve the selective uptake by thyroid follicular cells.

In addition to the in vitro experiments, we conducted in vivo studies to assess PSAPI accumulation in organs. First, we validated the affinity of the human TSHR antibody conjugated to PSAPI for murine tissue and confirmed its ability to recognize receptors on murine thyroid cells (Figure S10, Supporting Information). Following validation, PSAPI was administered to mice, and major organs were harvested 24 h post‐injection.

As shown in Figure 4f and g, PSAPI exhibited significant accumulation in the thyroid, whereas PSP, which lacks the TSHR antibody and iodine, showed no accumulation in the thyroid. Some PSAPI signal was observed in the kidney and liver, which is expected given their renal and hepatobiliary clearance pathways.^[^ ^44^ ^]^ These in vitro and in vivo results collectively demonstrate the thyroid‐targeting capabilities of PSAPI.

### 2.5 Enhanced HIFU Efficacy by PSAPI In Vitro

**Figure 5:**
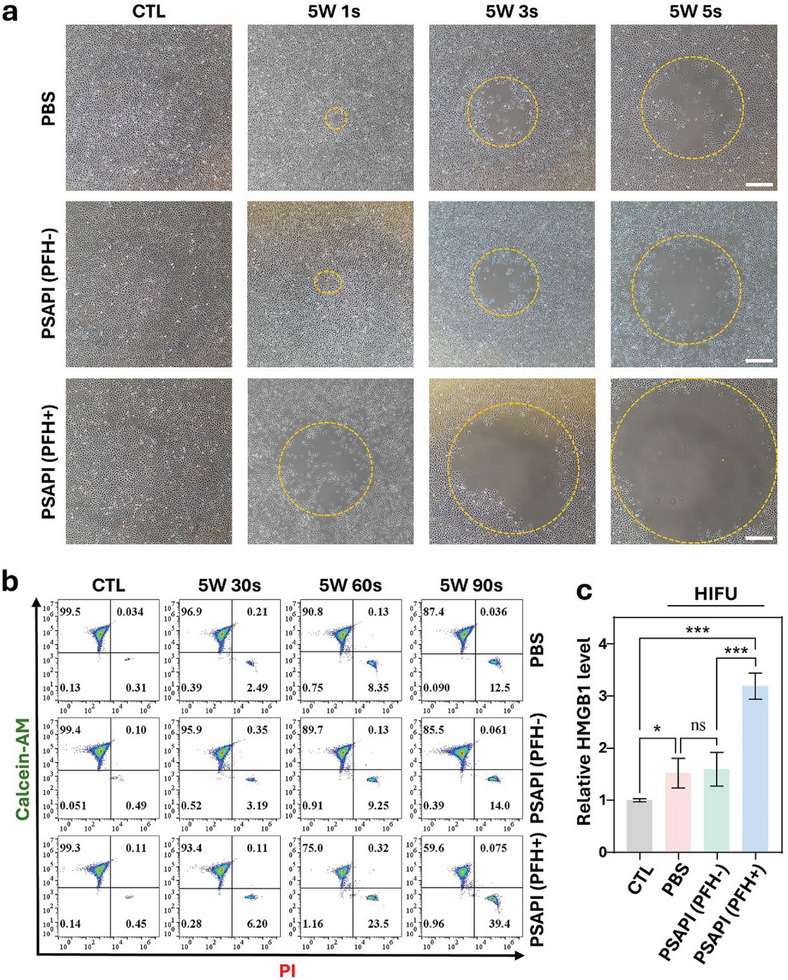
PSAPI enhanced HIFU ablation effect in vitro. a) Images of Nthy‐ori 3‐1 cells under HIFU exposure at 5 W for 1, 3, and 5 s. Cells were incubation with PBS, PSAPI (PFH‐), or PSAPI (PFH+) (125 µg mL⁻¹). b) Flow cytometric analysis of cells after HIFU treatment (5 W) for 30, 60, and 90 s. c) Relative HMGB1 levels in the supernatant of cells incubated with PBS, PSAPI (PFH‐), or PSAPI (PFH+) after HIFU radiation. Data are presented as mean ± SD (n ≥ 3). Statistical significance was determined using one‐way ANOVA followed by Dunnett's multiple comparison test. ns, not significant, ^*^ *p* < 0.05 and ^***^ *p* < 0.001 versus CTL. Scale bar: 500 µm.

The ability of PSAPI to enhance the efficacy of HIFU was first evaluated in vitro. And the role of PFH was assessed. As shown in **Figure** **5**a, HIFU‐induced detachment of adherent cells was significantly amplified with PSAPI treatment but not with PSAPI (PFH‐). We next applied HIFU to suspended cells and assessed cell death via flow cytometry. At a fixed HIFU power (5 W), increasing the irradiation time led to a rise in cell death from 2–3% (30 s) to 12–14% (90 s) in the saline‐treated and PSAPI (PFH‐) group. In comparison, PSAPI (PFH+) treatment enhanced cell death threefold (39.4% vs 12.5%) after 90 s of irradiation (Figure 5b), demonstrating PSAPI's exceptional ability to potentiate HIFU‐induced cell death.

The underlying mechanism of HIFU ablation is the induction of coagulative necrosis in tissues through ultrasound. Thus, the efficacy of ablation can be quantitatively assessed by the extent and severity of necrosis. To this end, we measured the release of the necrotic marker High‐Mobility Group Box 1 (HMGB1) from cells post‐HIFU irradiation. Thyroid cells treated with PSAPI (PFH+) exhibited significantly higher levels of HMGB1 release compared to PBS or PSAPI (PFH‐) group, indicating an increased degree of necrosis (Figure 5c). These results suggest that by encapsulating PFH, PSAPI effectively enhances HIFU efficacy.

### 2.6 Molecular Mechanisms of PSAPI Assisted HIFU Ablation

**Figure 6:**
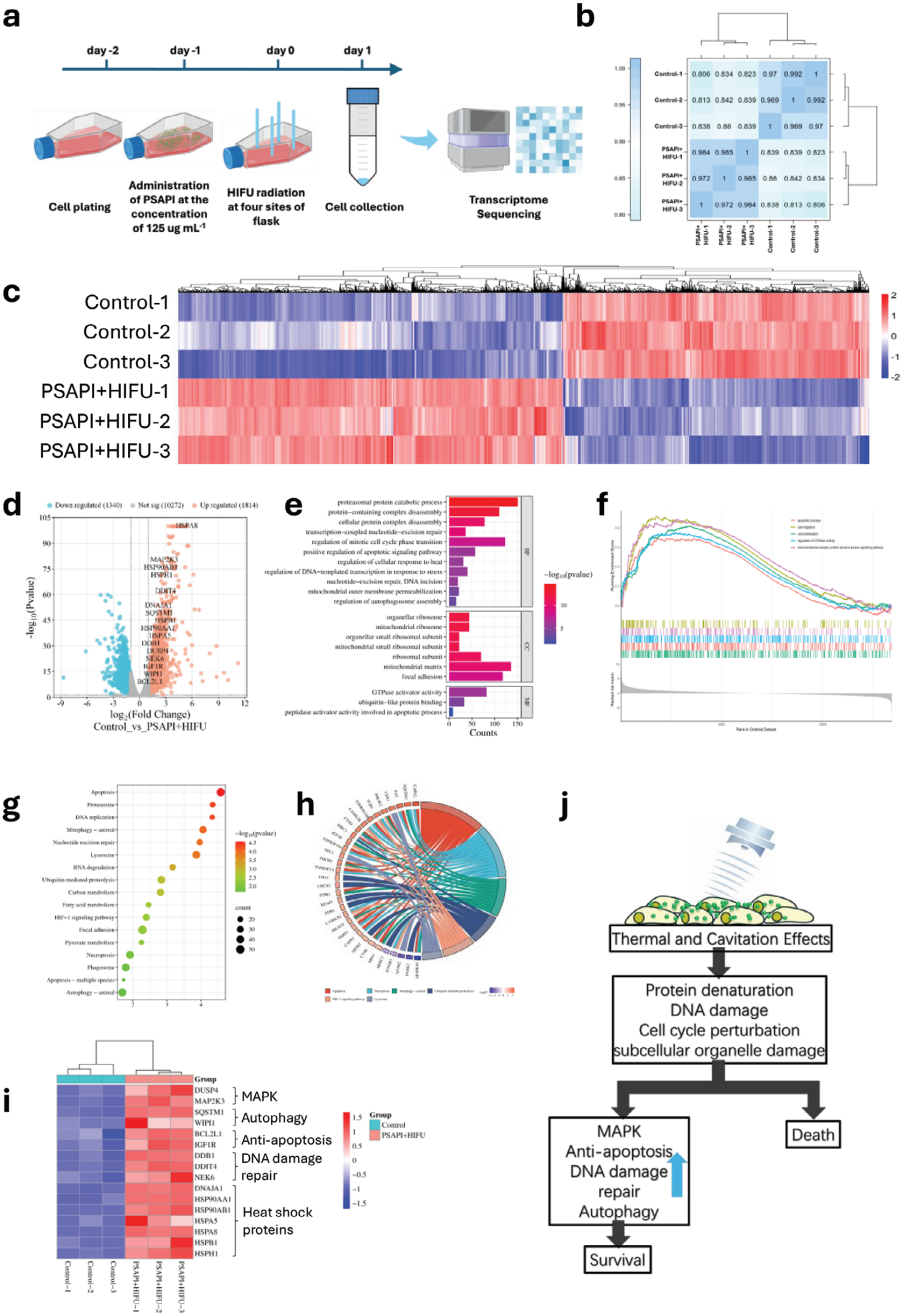
RNA‐Seq transcriptome analysis of HIFU radiation on PSAPI treated thyroid cells. a) Schematic illustration of the experimental design. b) Pairwise Pearson's correlation coefficients. c) Heat map of DEGs’ signature. d) Volcano plot of identified up‐regulated and down‐regulated genes. e) Gene ontology (GO) enrichment analysis. f) Gene set enrichment analysis (GSEA) for the significantly differential GO terms. g) The significantly enriched Kyoto encyclopedia of genes and genomes (KEGG) pathways. h) Chord chart of detailed correlations between up‐regulated DEGs and enriched KEGG pathways. i) Identified cell damage repair related genes. j) Proposed mechanism of cell response to PSAPI enhanced HIFU ablation.

To further elucidate the molecular mechanisms underlying HIFU and PSAPI‐induced cell damage and death, we performed transcriptome sequencing (RNA‐Seq) to quantify gene expression changes following HIFU irradiation. The experimental design is illustrated in **Figure** **6**a. As part of quality control, the pearson's correlation coefficient (r) among biological replicates in each group was close to 1, indicating strong correlations within treatment groups (Figure 6b). The analysis identified 3154 differentially expressed genes (DEGs), with 1814 upregulated and 1340 downregulated (Figure 6c,d).

Gene ontology (GO) enrichment analysis revealed that DEGs were significantly enriched in 11 biological process GO terms, including protein degradation, apoptosis, heat and stress responses, cell cycle regulation, DNA damage, and autophagy which reflect cellular responses to stress and the initiation of apoptosis and repair processes. In terms of cellular component, the DEGs were predominantly associated with the ribosome, mitochondria, and focal adhesions. At the molecular function level, DEGs were linked to GTPase activity, ubiquitin‐like protein binding, and apoptotic processes (Figure 6e). Gene set enrichment analysis (GSEA) further revealed upregulation of genes involved in apoptosis, cell migration, proliferation, GTPase activity, and transmembrane receptor protein tyrosine kinase signaling pathways (Figure 6f).

Kyoto encyclopedia of genes and genomes (KEGG) pathway analysis revealed that the DEGs were associated with key signaling pathways related to apoptosis, the proteasome, DNA replication, mitophagy, and nucleotide excision repair (Figure 6g). The top DEGs associated with these KEGG pathways are displayed in Figure 6h. The RNA‐seq analysis demonstrated that following HIFU irradiation, PSAPI‐treated thyroid cells experienced considerable stress induced by both thermal and cavitation effects. Heat induced by thermal effects denatures protein^[^ ^45^, ^46^ ^]^ and damages DNA.^[^ ^47^ ^]^ To address this, cells actively upregulated genes related to protein degradation and DNA damage repair. The cavitation of ultrasound causes cell lysis, deformation, and subcellular organelle damage, primarily to mitochondria.^[^ ^48^, ^49^ ^]^ Cell lysis, a complete destruction of cells, cannot be reflected by RNA‐seq data. Cell deformation affects cell cycle^[^ ^50^ ^]^ which was observed in DEGs rich in cell cycle regulation. Responded to mitochondrial and other subcellular organelles damage, cells initiated mitophagy and autophagy to clear damaged organelles. We identified 16 key genes involved in these damage‐repair mechanisms that were notably upregulated. As illustrated in Figure 6i, Mitogen‐activated protein kinases (MAPKs) are protein Ser/Thr kinases that mediate a broad range of cellular responses to external stimuli. The upregulation of critical MAPK‐related genes suggests that cells convert HIFU‐induced stress into internal cellular signaling pathways that coordinate survival mechanism. Heat shock proteins, which respond to thermal stress, were also elevated. As a result, cells actively engaged in self‐restoration by upregulating genes involved in autophagy, anti‐apoptotic mechanisms, and DNA damage repair. These key genes were further validated by mouse and rabbit models, providing robust evidence of the cellular response to HIFU‐induced stress and repair mechanisms. (Figures S13 and S14, Supporting Information).

In order to survive, cells orchestrated numerous signaling pathways to fight against insult and injury (Figure 6j). In the context of cancer‐related therapy, these damage‐control processes, if successful, could contribute to cancer survival and metastasis, potentially leading to more aggressive disease. The genes identified in this study may serve as important targets to further enhance the therapeutic efficacy of HIFU and broaden the application of our HIFU nano‐enhancer to thyroid cancer treatment.

### 2.7 In Vivo Ablation Efficacy and Biocompatibility

**Figure 7:**
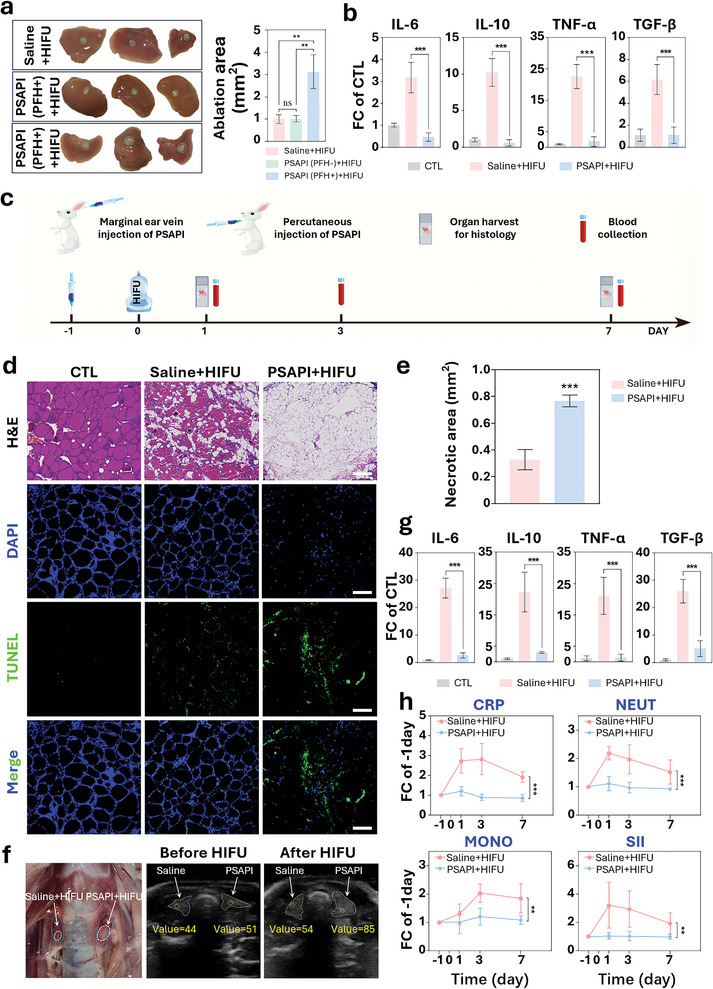
Effect of PSAPI on HIFU ablation in vivo. a) Photos and quantitative analysis of murine liver ablation area. Murine liver was harvested 24 h after PSAPI (PFH‐), PSAPI (PFH+), or saline tail vein injection and then treated by HIFU (5 W, 3 s). b) RT‐qPCR analysis of inflammatory cytokines’ level in murine livers. Mice were treated with HIFU (5 W, 60 s) at upper right quadrant of the abdomen where the liver located. Liver was harvested 24 h after treatment. c) Schematic illustration for experimental design. Rabbit was injected with PSAPI (4 mg kg^−1^) or saline percutaneously at thyroid or through marginal ear vein on day‐1. HIFU radiation (5 W, 300 s) was applied at day 0. Blood sample was collected at day ‐1, 1, 3, and 7. Organs were harvested at day 1 and 7 for pathological section. d) Representative H&E, DAPI, and TUNEL staining of rabbit thyroid section. e) Quantitative analysis of the necrotic area. f) Dissection photos and B‐mode ultrasound images of rabbit thyroid tissue before and after HIFU radiation. g) RT‐qPCR analysis of inflammatory cytokines’ level of rabbit thyroid after HIFU treatment. h) Serum inflammatory markers of rabbits. Data are presented as mean ± SD (n ≥ 3). Statistical significance was determined using t test, one‐way ANOVA followed by Dunnett's multiple comparison test or two‐way ANOVA followed by Bonferroni multiple comparison test. ns, not significant, ^**^ *p* < 0.01, and ^***^ *p* < 0.001. Scale bar: 100 µm.

At last, we investigated the potential of PSAPI to enhance the efficacy of HIFU ablation in vivo. Given that PSAPI retention was observed in the liver 24 h post tail vein injection in mice, we first conducted HIFU ablation on the murine liver. As shown in **Figure** **7**a, the ablation area in the PSAPI‐treated group was significantly larger compared to the saline‐treated control or PSAPI (PFH‐)‐treated group indicating the enhancement effect of PSAPI on HIFU ablation in which PFH plays an important role. The release of DCs from the disrupted PSAPI led to a marked reduction in pro‐inflammatory cytokines, suggesting a lower level of inflammation (Figure 7b).

Due to the small size of the murine thyroid, which precludes effective ablation studies, we employed a rabbit model for thyroid ablation. We first applied HIFU ablation to pork tissue to ensure the correct settings for rabbit thyroid. The temperature rise curve showed that after irradiating at 5 W for 300 s, the temperature of the ablated area reached up to 70 °C, which is above the phase transition temperature of PFH and thus sufficient to trigger the explosion of the nano‐bombs (Figure S11a, Supporting Information). As shown in Figure S12 (Supporting Information), the ablation depth and size were within the range of rabbit thyroid. Therefore, the operating parameters (Figure S11b, Supporting Information) employed were well‐suited for rabbit thyroid ablation. Human TSHR antibody affinity for rabbit thyroid tissue was confirmed as well (Figure S10, Supporting Information).

Displayed in Figure 7f, B‐mode ultrasound images showed that PSAPI drastically increased gray value of thyroid tissue after HIFU treatment (from 51 to 85) whereas saline treatment showed moderate elevation (from 44 to 54). Post‐therapy dissection images of rabbit thyroids showed an enlarged ablation area in PSAPI injected thyroid which is consistent with the results observed in mouse liver (Figure 7f). Quantification of necrotic area from H&E stained rabbit thyroid tissue revealed a substantial increase in coagulative necrosis in PSAPI treated group (Figure 7e). Furthermore, intact nuclear staining was still evident in H&E images of the saline group, indicating incomplete ablation, whereas almost all nuclear staining was absent in the PSAPI group, reflecting complete destruction of thyroid tissue (Figure 7d). Terminal deoxynucleotidyl transferase dUTP nick‐end labeling (TUNEL) staining also revealed a higher degree of DNA damage in PSAPI group (Figure 7d). Collectively, these findings demonstrate that PSAPI significantly enhances HIFU‐mediated ablation in thyroid tissue in vivo, leading to greater tissue destruction, increased necrosis, and higher levels of DNA damage.

To assess systemic inflammatory responses, we monitored inflammatory markers in rabbit blood before and after HIFU treatment. The saline group exhibited a significant increase in all markers including c‐reactive protein (CRP), neutrophils (NEUT), monocytes (MONO), and systemic immune‐inflammation index (SII), whereas the PSAPI group showed only minor increases (Figure 7h). In addition, pro‐inflammatory cytokine levels in thyroid tissue were significantly reduced in the PSAPI‐treated group (Figure 7g). A blind evaluation of post‐HIFU neck swelling was performed by independent evaluators, with the PSAPI‐treated rabbits showing significantly lower rabbit neck swelling index (RNSI) scores, indicating that PSAPI effectively mitigated post‐ablation edema (Figure S15, Supporting Information). Therefore, PSAPI not only enhances the therapeutic efficacy of HIFU ablation but also significantly reduces the inflammatory response following treatment.

The in vivo biocompatibility of PSAPI was evaluated through both mouse and rabbit models. Body weight remained stable following PSAPI injection and HIFU radiation (Figures S16 and S18, Supporting Information). H&E staining revealed no significant pathological damage to major organs, including the heart, liver, spleen, kidney, and lungs, when compared to the saline or control groups (Figures S17 and S19, Supporting Information). Routine blood tests including counting for white, red blood cells and platelets revealed no significant differences between the control, saline or PSAPI injection groups (Figure S20, Supporting Information). Blood biochemistry tests assessing liver and renal function in mice detected no significant differences (Figure S21, Supporting Information) and C‐reactive protein (CRP) levels were also stable among control, saline, and PSAPI groups (Figure S22, Supporting Information). These results suggest that PSAPI exhibits excellent biocompatibility, highlighting its potential for clinical applications.

## 3 Conclusion

To address the increasing demand for high‐quality healthcare, extracorporeal therapeutic techniques such as high‐intensity focused ultrasound (HIFU) provide safer and better alternatives, particularly for diseases like Graves' disease (GD), which is free of metastatic risk. This study aims to enhance the therapeutic efficacy of HIFU in GD and mitigate post‐operative complications, specifically edema, by developing a novel nanosystem—PSAPI. This system is designed to deliver the bubble‐generating agent (PFH) and the anti‐inflammatory drug (DC) to the thyroid. Both agents are encapsulated within a PLGA and silica shell which is further functionalized with TSHR antibodies to enable precise thyroid targeting. A PDA coating was applied to improve the biocompatibility of the NPs and facilitate iodine loading, further enhancing the system's specificity for thyroid tissue.

The design of PSAPI was meticulously optimized to ensure NP stability, good biocompatibility and proper functioning. Both in vitro and in vivo experiments demonstrated that PSAPI effectively targets thyroid tissue, enhances ablation efficiency, and significantly reduces the inflammatory response. These findings suggest that PSAPI represents a promising adjuvant for HIFU in the treatment of GD. Additionally, we elucidated the underlying signaling pathways and identified critical cellular damage‐repair genes involved in PSAPI‐assisted HIFU therapy. These genes may serve as potential therapeutic targets to inhibit cellular survival pathways post‐ablation, thereby maximizing the efficacy of HIFU in future studies.

## 4 Experimental Section

### Statistical Analysis

Experiments were conducted with at least three independent replicates (n = 3), and data were calculated and analyzed using GraphPad Prism 10. The results were expressed as mean ± standard deviation (SD). To compare two groups, unpaired Student's t‐tests were performed. For three or more groups, one‐way ANOVA was used followed by Dunnett's multiple comparison test. For two‐way comparisons, two‐way ANOVA was performed followed by Bonferroni's multiple comparison test. Significance levels are denoted as not significant (ns), *p < 0.05, **p < 0.01, and ***p < 0.001.

### Animal Ethics Statement

Murine studies were conducted in accordance with the regulations set forth by the Institutional Animal Care and Use Committee of Shantou University Medical College and approved by the Ethics Review Committee for Animal Welfare in Experiments (SUMCSY2024‐009). Rabbit studies were carried out in compliance with the protocols approved by the Institutional Animal Care and Use Committee of Shenzhen Huateng Biomedical Technology Co., Ltd., and authorized by the Ethics Review Committee for Animal Welfare (B202407‐4).

## Conflict of Interest

The authors declare no conflict of interest.

## Supporting information



Supporting Information

Supplemental Video 1

## Data Availability

The data that support the findings of this study are available from the corresponding author upon reasonable request.
