# Supplementary material for: Thyroid‐Targeted Nano‐Bombs Empower HIFU for Graves' Disease
Source: Adv Sci (Weinh). 2025 Jan 22;12(11):2414597. doi: 10.1002/advs.202414597 (PMC11923863; doi:10.1002/advs.202414597)
Supplement: Supplementary file 1 — Supporting Information [file ADVS-12-2414597-s002.docx]

Supporting Information

Thyroid-targeted Nano-bomb Empower HIFU for Graves' Disease

*Binhao Wang^1^, Zhuobing Yin^1^, Xiangyue You^1^, Hanwei Peng^1^* and Ying Jiang^1^**

^1^The Department of Head and Neck Surgery, Cancer Hospital of Shantou University Medical College, Shantou, Guangdong, 515041, P. R. China

Email: hwpeng@stu.edu.cn; yingjiang@stu.edu.cn

**1. Materials and methods**

**Materials**

Poly(lactide-co-glycolide) (PLGA, LA:GA=50:50, molecular weight 30,000-60,000) was purchased from Sigma-Aldrich (St. Louis, MO, USA). Dimethyl sulfoxide (DMSO), hematoxylin and eosin, Triton X-100 was purchased from Solarbio (Beijing, China). Dichloromethane (CH_2_Cl_2_), perfluorohexane (PFH), diclofenac (DC), Ethanol, didodecyldimethylammonium bromide (DDAB), (3-aminopropyl)triethoxysilane (APTES), tetraethyl orthosilicate (TEOS), ammonia solution, dopamine hydrochloride, tris(hydroxymethyl)aminomethane (Tris), Morpholineethanesulfonic acid (MES), sodium iodide, Cy5 NHS ester, Cy7 NHS ester, and 2*N*-(morpholino)ethanesulfonic acid (MES) were obtained from Aladdin (Shanghai, China). Cy2 NHS ester was purchased from Duofluor Inc (Wu han, China). Fetal bovine serum (FBS) was purchased from Vazyme (Nan jing, China). RPMI-1640 medium and 2-mercaptoethanol were purchased from Gibco (Carlsbad, CA, USA). Phosphate buffered saline (PBS), TUNEL apoptosis detection kit, cDNA Synthesis Kit, Green Master Mix, bovine serum albumin (BSA), penicillin-streptomycin solution and trypsin-EDTA solution were purchased from Yeasen (Shanghai, China). TSHR antibody (3B12) and horseradish peroxidase-conjugated secondary antibody were purchased from Santa Cruz Biotechnology (Dallas, TX, USA). NIS antibody was purchased from ABclonal (Wuhan, China). *N*-hydroxysuccinimide (NHS), 1-Ethyl-3-(3-dimethylaminopropyl)carbodiimide hydrochloride (EDC), Alexa Fluor™ 488 Phalloidin, ProLong™ Gold Antifade Mountant, Wheat Germ Agglutinin (WGA)-Alexa Fluor 647 was purchased from Thermo Fisher Scientific (Waltham, MA, USA). Cell Counting Kit-8 (CCK-8), Calcein AM/PI Cell Viability/Cytotoxicity Assay Kit, Alexa Fluor 488-labeled Goat Anti-Mouse IgG(H+L), Sodium Citrate Antigen Retrieval Buffer, ECL Chemiluminescence Detection Kit and BCA protein assay kit were purchased from Beyotime (Shanghai, China). 4% paraformaldehyde was purchased from Boster (Wuhan, China). Human High Mobility group protein B1 (HMGB-1) ELISA kit was purchased from Elabscience (Wuhan, China). TRIzol RNA kit were purchased from Ncmbio (Suzhou, China). Nthy-ori 3-1 cell, HEK-293T cell, THP-1 cell and BT-549 cell were purchased from Procell (Wuhan, China). NCM460 cell was purchased from Zqxzbio (Shanghai, China). Huvec cell line was purchased from Stemrecell (Shanghai, China). KM mice were purchased from Zhuhai BesTest Bio-Tech Co (Zhuhai, China). The New Zealand rabbits were purchased from Wuwei Biotechnology (Jiangsu, China).

**Characterization**

The morphology was visualized using a Thermo Scientific Verios 5 UC scanning electron microscope (SEM). Transmission electron microscopy (TEM) images and energy-dispersive X-ray spectroscopy (EDS) analysis were performed using a JEM-F200 TEM. Particle size, dynamic light scattering (DLS) profiles, and zeta potential were measured using a Zetasizer Pro (Malvern Panalytical). UV-vis spectra were recorded on a PerkinElmer LAMBDA 850+ spectrophotometer. Fourier transform infrared spectroscopy (FTIR) spectra were obtained using a Bruker TENSOR II spectrometer.

Nanoparticles (NPs) were dispersed and homogenized using a CJ-040SD ultrasonic cleaner. The absorbance in 96-well plates was measured using a TECAN microplate reader. Confocal fluorescence images were acquired using a ZEISS LSM800 upright laser scanning confocal microscope and a ZEISS LSM880 inverted laser scanning confocal microscope (live-cell workstation). Flow cytometry was performed using a BD Accuri™ C6 flow cytometer. B-mode ultrasound imaging was conducted using a Mindray MYLAB30 ultrasound system. RNA concentrations were measured using a NanoDrop 2000 spectrophotometer (Thermo Fisher Scientific). Polymerase chain reaction (PCR) was performed using a MiniAmp Plus Thermal Cycler (Thermo Fisher Scientific) and a C1000 Thermal Cycler (Bio-Rad). The PRO 5G-A ultrasound therapy device was provided by Shenzhen PRO HITU Medical Co., Ltd. (Shenzhen, China).

**Preparation of PD-PLGA (PP)**

50 mg of PLGA was completely dissolved in 2 mL of CH_2_Cl_2_ within a glass test tube. Subsequently, 10 mg of DC, dissolved in DMSO (100 µL), and 100 µL of PFH was added into the CH_2_Cl_2_ and mixed by an ultrasonic cell disruptor (800 W for 10 s) on ice bath. Following this, 4 mL of DDAB solution (0.1% w/v) was added to the mixture and subjected to sonication using the ultrasonic cell disruptor (800 W for 60 s) on ice bath to form an emulsion. The emulsion was then diluted with 40 mL of DDAB solution (0.1% w/v) and stirred (500 rpm for 3 h) to facilitate the evaporation of CH_2_Cl_2_. After centrifugation at 10,000 rpm for 10 min at 4 ℃ and washed with DDW (Double Distilled Water) twice, PP was obtained. The preparation process of non-PFH PP was similar to that of PFH-loaded PP, with the only difference being that PFH was not included during DC and PLGA mixing procedure.

**Preparation of PD-PLGA@Si (PS)**

For synthesizing the silica shells, 100 mg of DDAB was dissolved in a solution comprising ethanol (10 mL), DDW (40 mL), and ammonia solution (0.5 mL, 25 wt%). Following stirring at RT for 30 min, TEOS (60 µL), APTES (60 µL) and PP (50 mg) were rapidly added into this solution with vigorous stirring (1,000 rpm) for 3 h at RT. Sequentially, the obtained PS was centrifuged at 10,000 rpm for 10 min at 4 ℃ and washed twice with DDW.

**Preparation of PD-PLGA@Si-Ab (PSA)**

The TSHR antibody conjugation with PS was performed using EDC/NHS chemistry. Initially, NHS (6 mg) and EDC (10 mg) were separately dissolved in 1 mL of MES buffer to prepare 50 mM solutions. Then, 4 µL of EDC solution and 10 µL of NHS solution were added to the lyophilized TSHR antibody, followed by 86 µL of 1× MES buffer, and the mixture was incubated at RT for 30 min. The reaction was quenched by adding 1.4 µL of 2-mercaptoethanol. Sequentially, PS was added to the mixture and incubated for 2 h at RT. The resulting PSA were then centrifuged at 10,000 rpm for 10 min at 4 ℃ and washed twice with DDW.

**Preparation of PD-PLGA@Si-Ab/PDA (PSAP**)

25 mg of PSA were incubated in 25 mL of Tris buffer (10 mM, pH 8.5) containing 0.04% w/v dopamine with stirring (500 rpm for 1 h) at RT. Then the mixture was centrifuged at 10,000 rpm for 10 min at 4 ℃ and washed twice with DDW to obtained PSAP.

**Preparation of PD-PLGA@Si-Ab/PDA-I (PSAPI)**

PSAP was resuspended in 10 mL of 0.1 M NaI solution and incubated for 60 min, with intermittent ultrasonication for 10 s every 10 min to prevent particle aggregation and ensure uniform exposure of the PSAP. Following incubation, the PSAPI were isolated by centrifugation at 10,000 rpm for 10 min at 4 °C and washed twice with DDW.

**In vitro DC release with or without HIFU irradiation**

50 mg of PSAPI was dispersed in 3 mL of PBS and then placed into a dialysis bag (Mw = 3,000). The dialysis bag was immersed in a beaker containing 197 mL of PBS, stirred magnetically at 4 °C. Measurements were taken at predetermined time points: 0, 1, 2, 4, 6, 8, 10, 12, and 14 days. At each time point, 3 mL of PBS was extracted from the beaker for UV-vis absorption measurements at the DC absorption peak of 275 nm. After each extraction, 3 mL of fresh PBS was added to the beaker to maintain a constant volume. To calculate the release rate of DC, the concentration of DC at each time point was determined using UV-vis absorption. The amount of DC released at each time point (M_released_(t)) was calculated by multiplying the concentration of DC (C(t)) by the volume of the sample taken. The cumulative amount of DC released was calculated by summing the concentrations measured at each time point and adjusting for the addition of fresh PBS. The formula used for calculating the cumulative released drug is:

$$M_{\mathrm{released}}(t)=\sum_{i=1}^{n} \left( C(t_{i})\times V_{\mathrm{sampled}} \right)+C(t_{n})\times V_{total}$$

Where n is the number of time points, ​V_sampled_ is the sampled volume (3 mL), and V_total_ is the total PBS volume (197 mL). The release rate R(t) was calculated as the percentage of the cumulative DC release relative to the total amount of DC loaded in the dialysis bag:

$$R(t)=\frac{M_{released}(t)}{M_{total}}\times100\%$$

Similarly, prepare a PSAPI or non-PFH PSAPI solution in PBS at a concentration of 1 mg mL^-1^. Then, 3 mL of each solution was placed into individual 15 mL centrifuge tubes. These NPs were then subjected to HIFU irradiation at power settings of 0, 3, 4, and 5 W for various durations: 0, 1, 3, 5, 10, 15, and 20 min. The focus of HIFU (focal length: 8 mm) was precisely located in the centrifuge tubes containing the NPs. After HIFU treatment, samples were centrifuged and the supernatant was then collected and followed by filtration using a 0.22 µm filter for UV-vis absorption measurements.

**Cell culture**

Nthy-ori 3-1 cells were cultured in RPMI-1640 medium with 10% FBS and penicillin-streptomycin solution at 37 °C in CO_2_ incubator (95% air and 5% CO_2_). BT-549 cells were cultured in RPMI-1640 medium with 10% FBS, 10μg mL^-1^ insulin and penicillin-streptomycin. THP-1 cells were cultured in RPMI-1640 medium with 10% FBS, 0.05 mM β-mercaptoethanol and penicillin-streptomycin. HEK-293T, Huvec and NCM460 cells were cultured DMEM medium with 10% FBS and penicillin-streptomycin.

**In vitro cytotoxicity**

To evaluate the cytotoxicity of NPs, Nthy-ori 3-1 cells, THP-1 cells, HEK-293T, Huvec cells were seeded in 96-well plates (1 × 10^4^ cells per well) or 6-well plates (1 × 10⁶ cells per well) and then cultured for 24 h. After attachment, the cells were incubated with various concentrations of PP, PS, PSP and PSAPI (0, 30, 60, 125, 250, 500 μg mL^-1^) for 24, 48, and 72 h. Following incubation, CCK-8 working solution or Calcein AM/PI working solution was added, and the plates were incubated for 2 h or 30 min at 37 °C accordingly. Absorbance of CCK-8 assay was measured at 450 nm by a microplate reader. The live/dead cell by for flow cytometry, after staining cells were trypsinized, collected, and resuspended in PBS for flow cytometry analysis.

**Cellular uptake**

NPs were first doped with fluorescent dyes by mixing with Cy2, Cy3 or Cy7 NHS ester for 2 h at RT accordingly. To evaluate the cellular uptake capability of NPs, Nthy-ori 3-1, BT-549 and NCM460 cells were seeded onto 14 mm round coverslips placed in 24-well plates at a density of 2 × 10⁴ cells per well and cultured for 24-48 h. After attachment, the cells were incubated with 1 mL of fresh medium containing Cy5-doped NPs (125 μg mL^-1^) for 24 h. Cells were then washed three times with PBS to remove excess NPs and prepared for flowcytometry or confocal microscopy. For flowcytometry, cells were stained with Wheat Germ Agglutinin (WGA)-Alexa Fluor 647 (10 μg mL^-1^) for 10 min at 37 °C and then collected, resuspended in PBS and tested by flow cytometer immediately. For confocal, cells were fixed with 4% paraformaldehyde for 15 min at RT, permeabilized with 0.1% Triton X-100 in PBS for 5 min, and then blocked with 1% BSA in PBS for 30 min at RT. After blocking, Alexa Fluor™ 488 Phalloidin was added and incubated for 20 min at RT to label F-actin. Finally, the cells were stained with DAPI for 10 min to label nuclei and washed three times with PBS. The coverslips were then mounted with ProLong™ Gold Antifade Mountant. Fluorescence images were captured using a ZEISS LSM800 confocal laser scanning microscope, followed by quantitative analysis of Cy5 fluorescence intensity per cell to assess the cellular uptake efficiency.

**Live cell uptake**

To monitor live cell uptake of PSAPI, confocal glass-bottom dish was drilled diagonally using syringe needle to create channels for particle flow. Heated 0.5% agarose solution in DMED was poured into the dish and allowed to solidify. Once solidified, the gel on top of glass bottom was carefully removed, while the surrounding gel was left intact to secure both the needle channels and the insertion of coverslip which create two chambers allowing the separate seeding of Nthy-ori 3-1 and NCM460 cells. After seeding for 24 h, the coverslip was removed, and the cells were co-cultured for another 24 h for better attachment. Cells were stained with WGA-Alexa Fluor 647 for better visualizing under confocal. Subsequently, Cy2 doped PSAPI was injected into the system via a syringe pump which ensures consistent and uniform flow at the rate of 100 μl min^-1^ through the channel created by syringe needles. Continuous imaging was performed using a ZEISS LSM880 confocal microscope over a period of 2 h, capturing images every minute to monitor the real-time live cell uptake of PSAPI.

The flow rate of PSAPI was determined according to blood flow rate inside thyroid under hyperthyroidism (15-197 mL min^-1^ per gram tissue)^[1]^. Approximately a million cells cover a glass-bottom dish weighed around 10^-3^ g^[2]^. The blood flow volume for 10^-3^ g tissue is 15-197 μL min^-1^. Thus, to mimic blood flow, PSAPI was injected at the speed of 100 μL min^-1^.

**In vivo imaging**

Adult female KM mice (25 to 30 g) were housed in groups of four per cage, with ad libitum access to chow and water. They were kept under temperature-controlled conditions on a 12-h light/dark cycle. To investigate the biodistribution of PSAPI, Cy5-doped NPs were injected via the tail vein into KM mice at a dose of 4 mg kg^-1^. The mice were on a low-iodine diet for two weeks before the experiment and fasted for 12 h prior to euthanasia. After 24 h post-injection, the mice were euthanized. Then, saline was used for cardiac perfusion to clear residual NPs from the bloodstream. The thyroid, along with major organs including the heart, liver, spleen, lungs, and kidneys, were carefully dissected and imaged using a spectrum imaging system. Fluorescence intensity was then quantitatively analyzed to evaluate NPs accumulation in each organ.

**Blood biochemistry and histopathology**

Biochemical and histopathological assessments were conducted at 1 d and 7 d post-injection. Mice without treatment served as the blank control group. Blood was collected via cardiac puncture and then allowed to stand for 2 h before being centrifuged at 3,000 rpm for 20 min to collect the serum which was used to measure hepatic and renal function markers such as alanine aminotransferase (ALT), glutamic oxaloacetic transaminase (AST), total bilirubin (TBIL), direct bilirubin (DBIL), blood urea nitrogen (BUN), creatinine (CREA), and uric acid (UA) detected by Servicebio (Wuhan, China). Whole blood was collected in EDTA anti-coagulant tubes for blood routine analysis, which included measurements of white blood cell count (WBC), lymphocyte percentage (Lymph%), monocyte percentage (Mon%), granulocyte percentage (Gran%), red blood cell count (RBC), hemoglobin (HGB), platelet count (PLT), and mean platelet volume (MPV), also analyzed by Servicebio (Wuhan, China). C-reactive protein (CRP) levels were measured using an ELISA kit from Servicebio (Wuhan, China).

Following blood collection, mice were euthanized, and major organs including heart, liver, spleen, lungs, kidneys, thyroid were harvested, fixed with paraformaldehyde, embedded in paraffin, and processed for histopathological examination using hematoxylin and eosin (H&E) staining.

**HIFU irradiation and temperature measurement**

To determine the system parameters, HIFU irradiation was first applied to pork tissue. A temperature probe was positioned at the focal point of the HIFU treatment to measure the temperature increase at the ablation center within the tissue. Temperature data were recorded at predetermined time intervals to generate a time-temperature curve illustrating the thermal effect during the ablation process. Following HIFU treatment, the pork tissue was carefully sliced to measure the distance from the deepest and shallowest points of ablation to the surface. These measurements were used to assess the rate of temperature elevation and the depth of ablation at the focal point.

**HIFU radiation in vitro**

To assess the PSAPI enhanced HIFU ablation effect in vitro, Nthy-ori 3-1cells incubated with PSAPI (PFH-), PSAPI (PFH+) or without PSAPI (125 μg mL^-1^ for 24 h) were exposed to HIFU with the probe suspended 8 mm above the well surface, ensuring that the focal point was precisely on the cells. HIFU exposures were conducted for 1, 3, and 5 s. Post-irradiation, cells were observed under a microscope.

For flowcytometry examination of live/dead cell, Nthy-ori 3-1cells incubated with PSAPI (PFH-), PSAPI (PFH+) or without PSAPI (125 μg mL^-1^ for 24 h) were resuspended in PBS and transferred to 15 mL centrifuge tubes before being exposed to HIFU at 5 W for 30, 60 and 90 s. Following HIFU treatment, cells were stained with Calcein AM/PI and subjected to flow cytometric analysis for quantitative evaluation.

**Transcriptome sequencing**

Nthy-ori 3-1 cells were seeded into T12.5 flasks and cultured until 80% confluence was reached. The cells were then incubated with 125 μg mL^-1^ of PSAPI for 24 h. Prior to HIFU treatment, the flasks were filled with complete culture medium and air bubbles were carefully eliminated. The flask caps were sealed with parafilm. Flasks were then placed in a water tank filled with degassed water and subjected to HIFU radiation at 5 W for 60 s at four distinct locations. Following HIFU treatment, the culture medium was collected and centrifuged at 1,000 rpm for 5 min to recover suspended cells, which were then returned to their respective flasks for further cultivation. After 24 h, cells from each sample were collected, washed once with PBS, and lysed using TRIzol reagent by gentle pipetting until the solution became clear. Samples were then frozen at -80 °C and shipped on dry ice to Beijing Tsingke Biotech Co. (Beijing, China) for transcriptomic sequencing.

**ELISA assays**

24 h after HIFU treatment, the levels of Human High Mobility Group Protein B1 (HMGB-1) in Nthy-ori 3-1 cells suspensions were measured using ELISA kits. Briefly, cell supernatant was collected by centrifugation at 3,000 rpm for 10 min. The supernatant was lyophilized and reconstituted with 100 µL of standard diluent which was added to a pre-coated 96-well plate and incubated at 37 °C for 90 min. After discarding the liquid, the plate was washed and followed by incubating with 100 µL of biotinylated anti-HMGB1 antibody for 1 h. After washing, 100 µL of HRP-conjugated enzyme was added and incubated at 37 °C for 30 min. And then 100 µL of TMB substrate was added to the plate which was incubated in the dark until a blue color developed. The reaction was stopped with 50 µL of stop solution, and absorbance was measured at 450 nm. HMGB1 concentrations were calculated from a standard curve.

**HIFU ablation in vivo**

New Zealand rabbits (2.5 to 3 kg) were housed individually in standard cages with ad libitum access to chow and water, under temperature-controlled conditions with a 12-h light/dark cycle. Rabbits were selected and stochastically assigned to three groups, with three rabbits per group: saline group, PSAPI group, and saline & PSAPI group (PSAPI percutaneously injected into the left thyroid lobe and saline into the right). The rabbits in the saline group and PSAPI group were administered PSAPI (4 mg kg⁻¹) or saline via ear marginal vein injection, respectively. The rabbits in saline & PSAPI group underwent percutaneous injection of PSAPI (2 mg mL^-1^, 50 µL) or saline (50 µL) was guided by B-mode ultrasound. 24 h following injection, the rabbit's thyroids were treated with HIFU at 5 W for 300 s. This procedure was conducted under B-mode ultrasound guidance to ensure precise localization of the thyroid gland. The saline & PSAPI group was subject to dissection and photographic documentation 24 h post-HIFU treatment, and thyroid tissues were collected for H&E staining and quantitative PCR (qPCR) analyses. Meanwhile, the body weights of rabbits in the saline group and PSAPI group were monitored daily for 7 d, and blood samples were drawn on days 1, 3, and 7 post-treatments to evaluate hematological parameters.

**Real-Time qRT-PCR**

Harvested organ tissues, weighing between 30 to 50 mg, were thoroughly ground and immediately transferred into TRIzol reagent to be lysed for total RNA extraction of total RNA using the TRnaZol RNA Kit. The purity and concentration of the isolated RNA were quantitatively assessed by NanoDrop 2000 spectrophotometer. The total RNA extracted was reverse transcribed into complementary DNA (cDNA) using the cDNA Synthesis Kit. This kit features a genomic DNA elimination step to prevent genomic DNA contamination in the PCR reactions. The reverse transcription was conducted using the MiniAmp Plus Thermal Cycler. The synthesized cDNA served as the template for qPCR, which was conducted using the C1000 Thermal Cycler (Bio-Rad) with the Green Master Mix. Primers specifically designed for the target genes were synthesized by Ruibiotech (Beijing, China). The sequences of all primers are shown in **Table S1** and **Table S2**.

**TUNEL apoptosis detection**

Organ tissues were harvested and processed into paraffin sections, followed by deparaffinization in xylene and rehydration through graded ethanol. A hydrophobic barrier was then applied around the tissue sections. Permeabilization was performed by incubating the sections with 100 μL of Proteinase K working solution at RT for 20 min, and then the sections were treated with equilibration buffer for 30 min. After removing the equilibration buffer, 50 μL of TdT incubation buffer was applied to each section, and the slides were incubated in a humidified chamber at 37 °C for 60 min in the dark. Post-incubation, the sections were washed three times with PBS. Nuclear counterstaining was performed with DAPI for 10 min. Fluorescence microscopy was employed for the analysis of the stained sections.

**Assessment of neck swelling in rabbits**

To assess the impact of PSAPI on neck swelling following HIFU treatment, visual assessments were conducted by independent evaluators. Photographs of each rabbit's neck were taken 24 h after the HIFU ablation to visually document the extent of swelling. To ensure unbiased assessment, three evaluators, blinded to the treatment each rabbit received, scored the severity based on the photographic evidence. The RNSI (rabbit neck swelling index), ranging from 0 (no swelling) to 5 (severe swelling), provided a subjective yet quantitative standard to assess swelling severity. Each photograph was independently assessed, and scores were compiled to calculate an average for each group.

**Immunofluorescence**

To validate the affinity of human TSHR antibody for murine and rabbit thyroid tissues, thyroid sections were subjected to immunofluorescence staining. Briefly, the paraffin sections were first deparaffinized in xylene and rehydrated through a series of graded alcohols to water. Antigen retrieval was then performed using a sodium citrate antigen retrieval buffer, heated in a water bath at 95 ℃ for 15 min to expose the TSHR epitopes. Subsequently, the sections were blocked with 2% BSA in PBS for 1 h at RT and followed by incubation with anti-TSHR antibody (diluted 1:200) at 4 ℃ overnight. After incubation, the sections were thoroughly washed with PBS. Alexa Fluor 488-labeled goat anti-mouse IgG (diluted 1:500) was then applied and incubated for 1 h at RT. Nuclear counterstaining was performed with DAPI for 10 min. The stained sections were then observed under a fluorescence microscope.

**Western blot**

Nthy-ori 3-1, BT-549 and MCN460 were collected and lysed on ice using RIPA buffer supplemented with a protease inhibitor cocktail to prevent protein degradation. The cell lysate was sonicated twice for 5 s each. The protein concentration was quantified using a BCA protein assay kit. After being prepared in sample buffer and boiled for 5 min, samples were then subjected to SDS-PAGE and transferred to a PVDF membrane. The membrane was blocked with 5% non-fat milk in TBST and incubated overnight at 4 °C with the TSHR antibody (1:500), NIS antibody (1:1000), or actin antibody (1:1000). After washing, the membrane was incubated with a horseradish peroxidase-conjugated secondary antibody (1:1000) for 1 h at RT. Proteins were visualized using ECL Chemiluminescence Detection Kit.

**2. Supporting Figures**

**a**

**b**


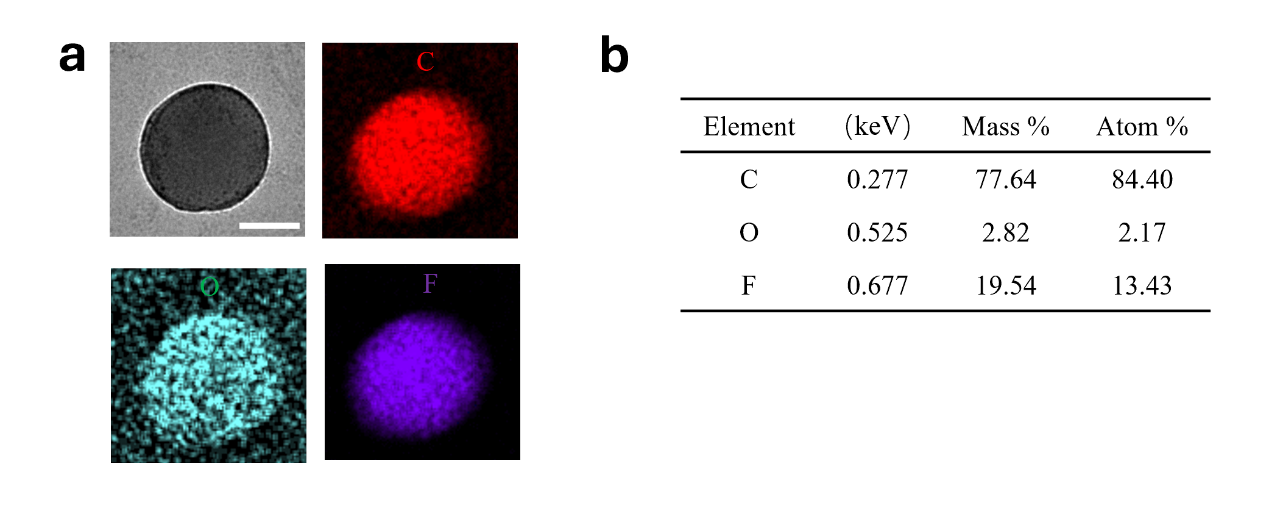

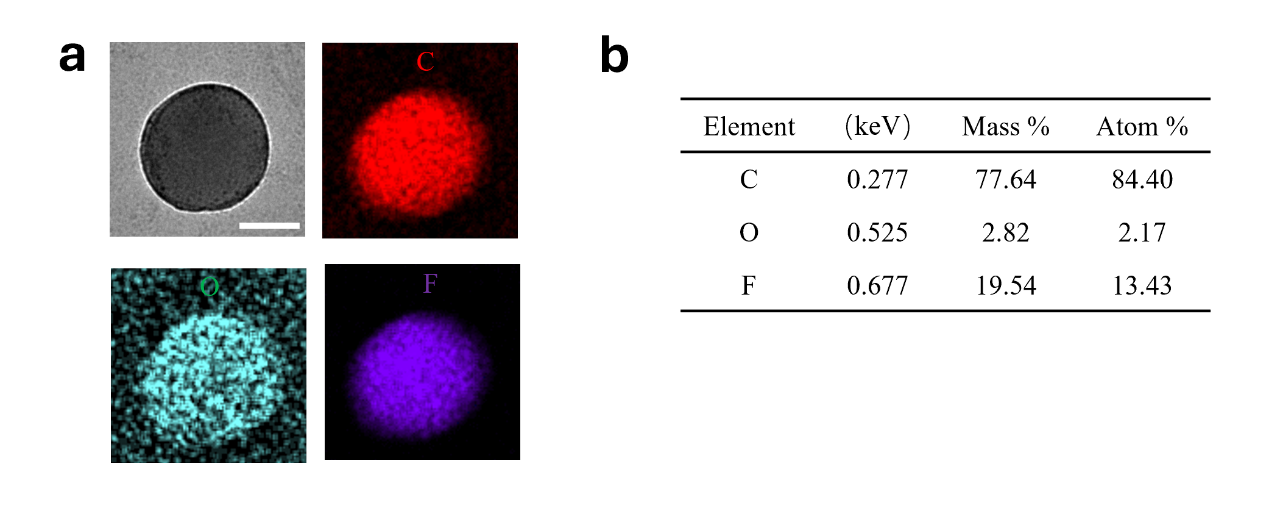


**Figure S1.** Characterization of PP. (a) TEM and EDS element mapping images. (b) EDS elemental analysis of PP. Scale bar: 100 nm.

**a**

**b**


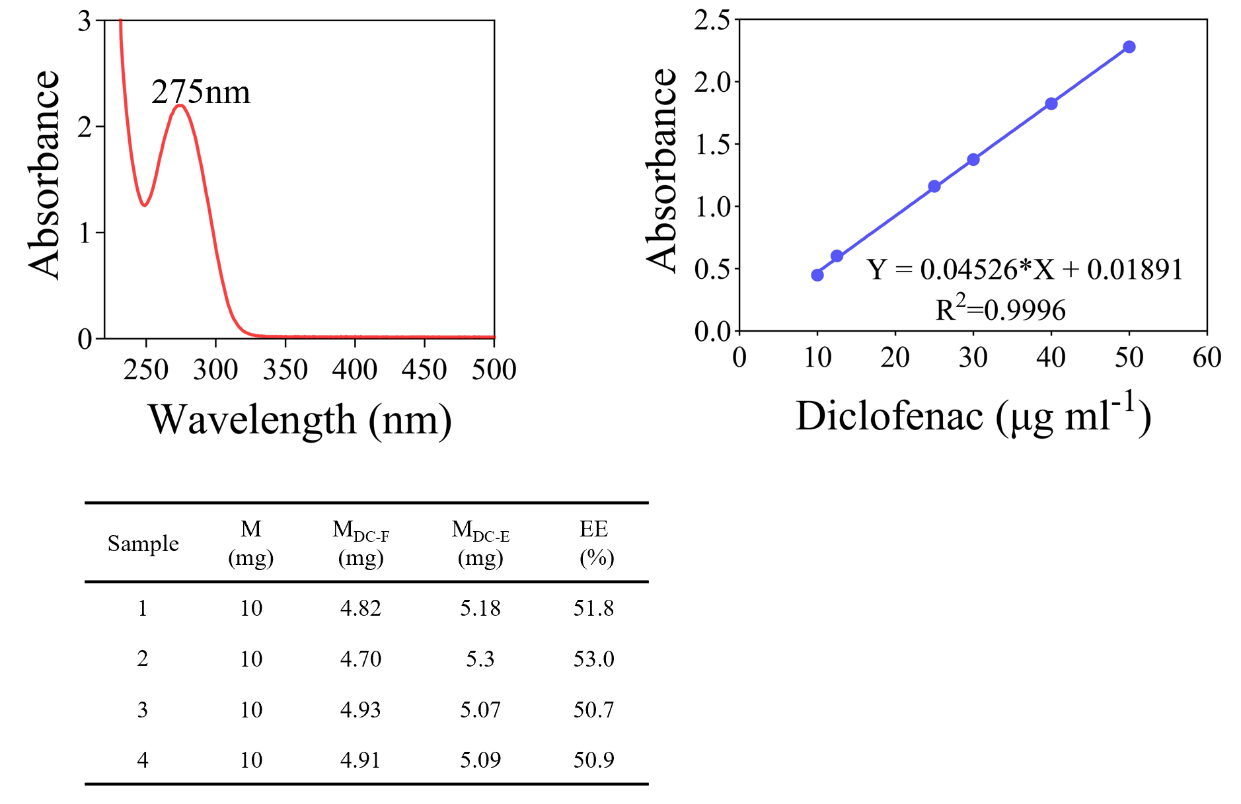


**c**

**Figure S2.** Diclofenac (DC) encapsulation efficiency. (a) UV spectra of DC. (b) DC Standard curve, a linear regression was deduced with equation of Y = 0.04526*X + 0.01891. (c) Calculated encapsulation efficiency (EE) of DC. M = mass of DC, M_DC-F_ = mass of free DC in supernatant, M_DC-E_ = mass of encapsulated DC.


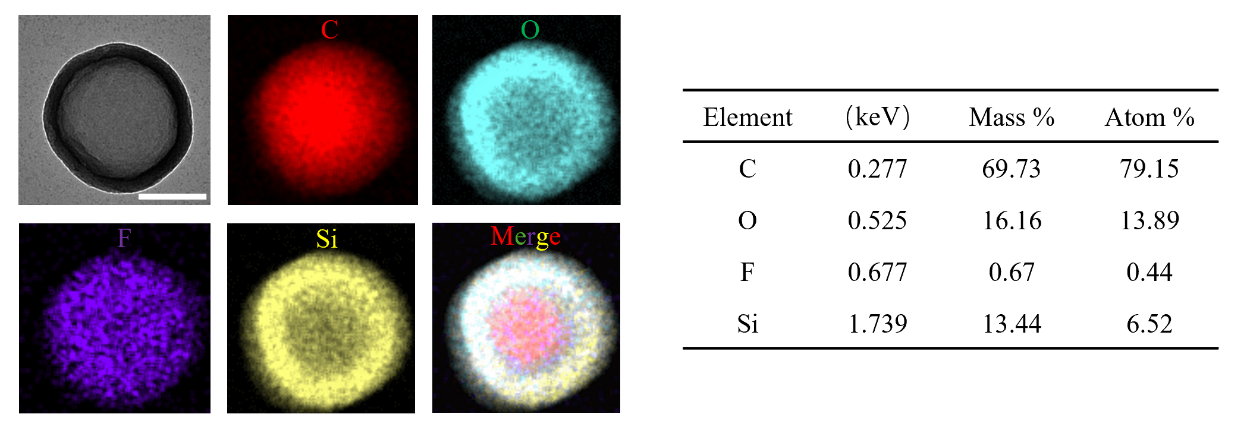


**b**

**a**

**Figure S3.** Characterization of PS. (a) TEM and EDS element mapping images. (b) EDS elemental analysis of PS. Scale bar: 100 nm.


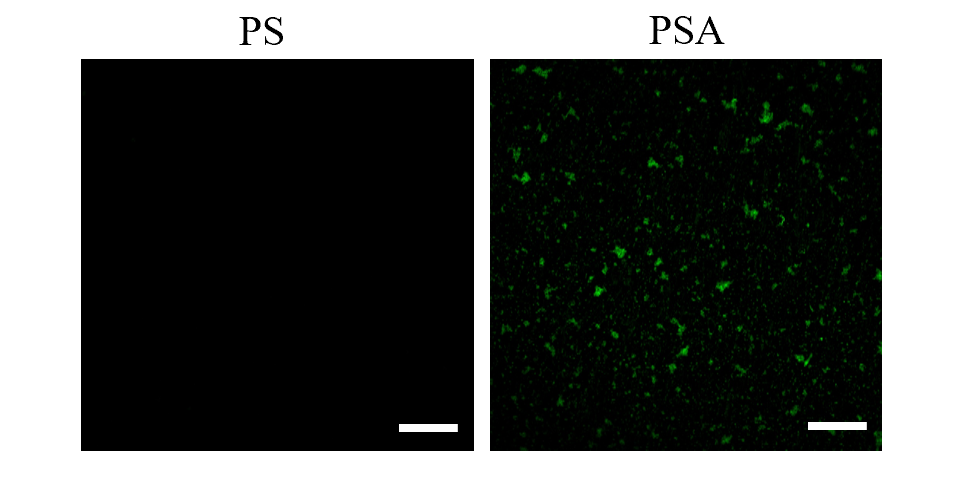


**Figure S4.** Antibody conjugation validation. PS and PSA were incubated with secondary fluorescent antibody for 2 h, washed 3 times with PBS and then visualized under fluorescent microscope. Scale bar: 20 μm.


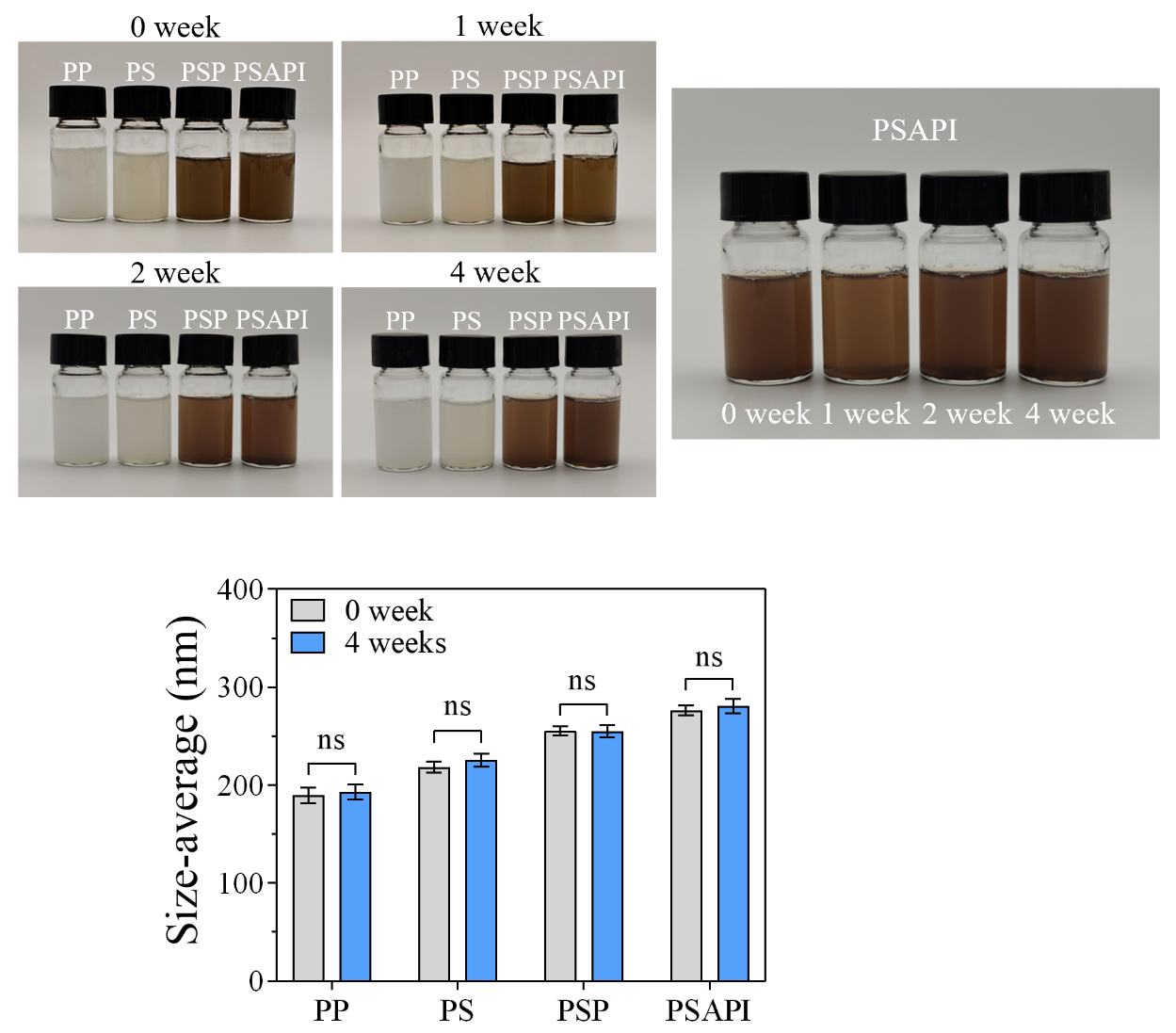


**a**

**b**

**Figure S5.** The stability of NPs. (a) Photos of NPs dispersed in PBS. (b) Average size of NPs at 0 and 4 weeks. Data are presented as mean ± SD (n = 3). Statistical significance was determined using t test. ns, not significant.


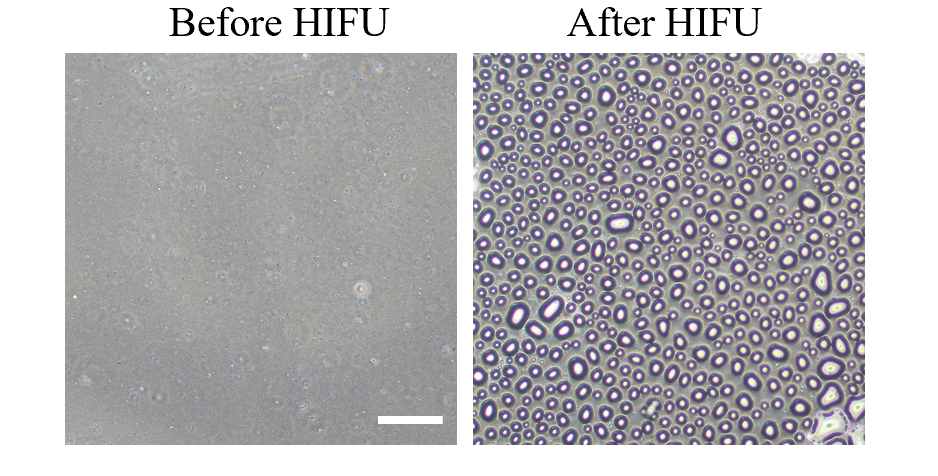


**Figure S6.** Images of PSAPI releasing bubble under heating at 60 °C for 300 s. Scale bar: 500 μm


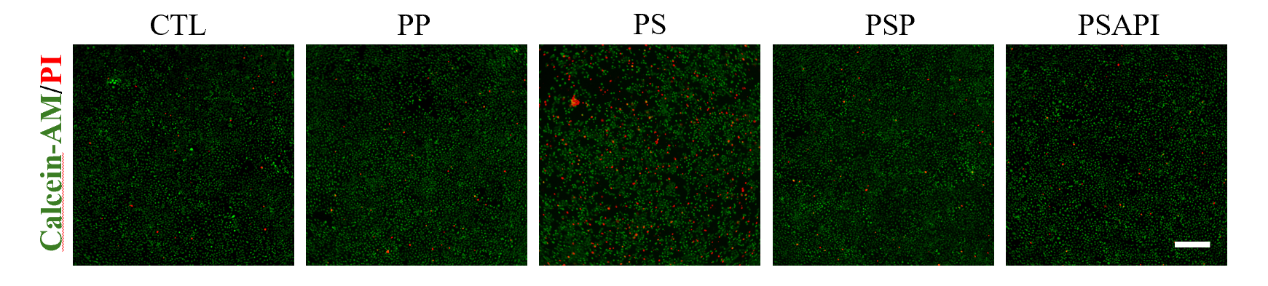


**Figure S7.** Fluorescent microscope images of live/dead stained Nthy-ori 3-1 cells after incubating with NPs at concentration of 125 µg mL⁻¹ for 24 h. Scale bar: 500 μm.


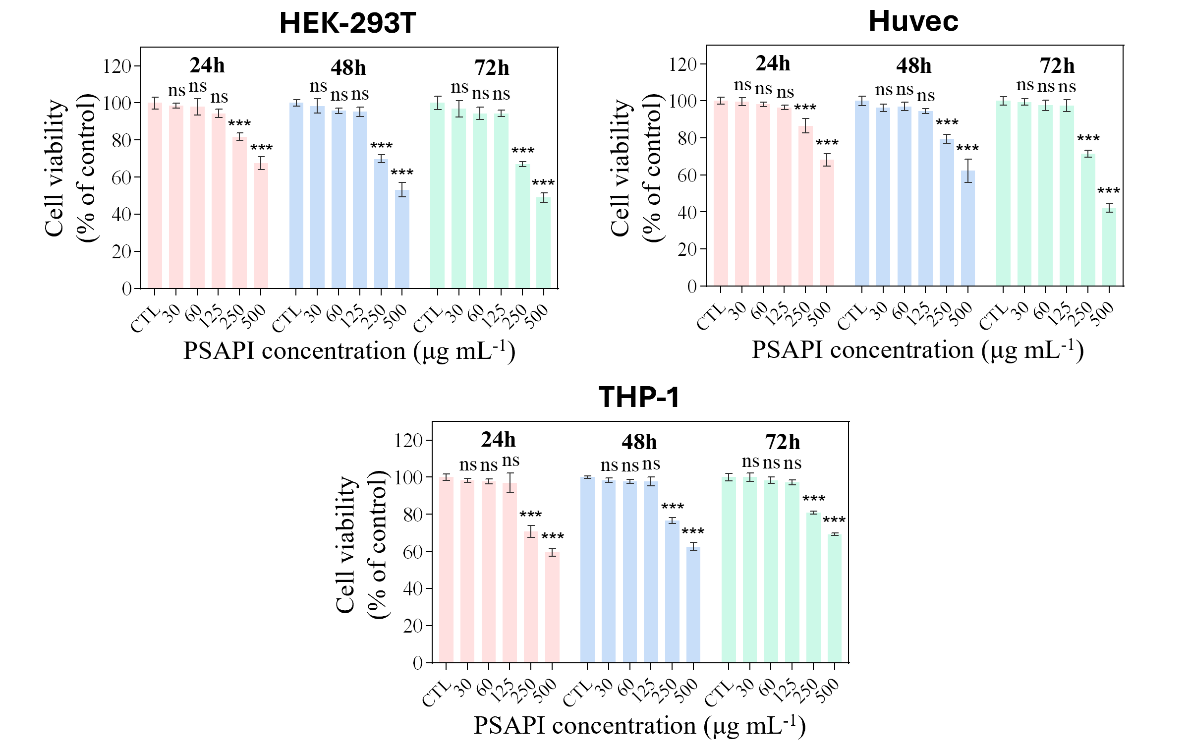


**Figure S8.** Relative cell viability of HEK-293T, HUVEC, and THP-1 cells following incubation with PSAPI at various concentrations for 24, 48, and 72 h. Data are presented as mean ± SD (n≥ 3). Statistical significance was determined using one-way ANOVA followed by Dunnett’s multiple comparison test. ns, not significant, and ***p < 0.001 versus CTL.


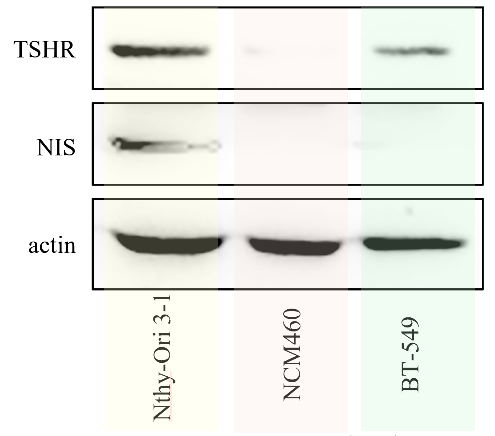


**Figure S9.** TSHR and NIS protein expression in Nthy-Ori 3-1, NCM460 and BT-549 cells.


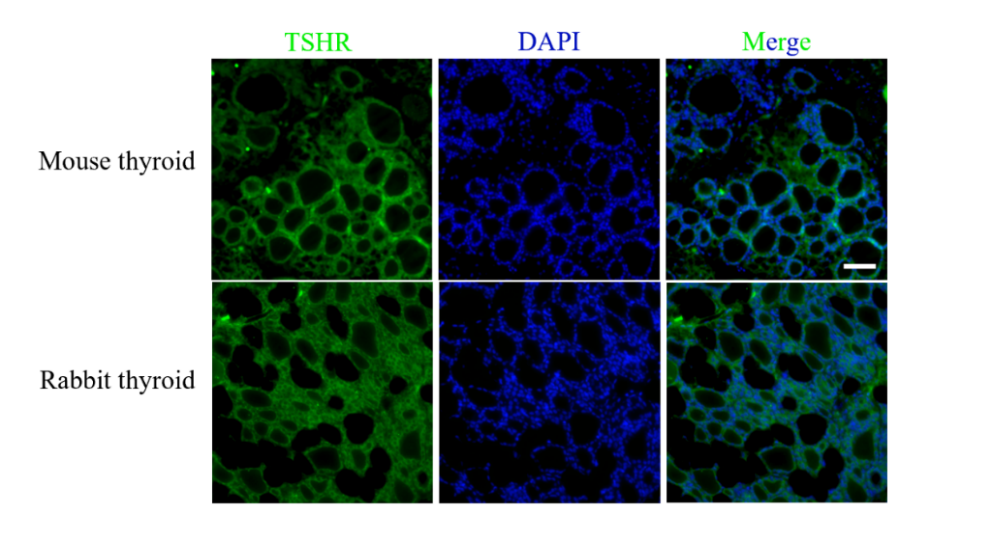


**Figure S10.** Antibody validation in animal tissue. Scale bar: 20 μm.

**a**

**b**


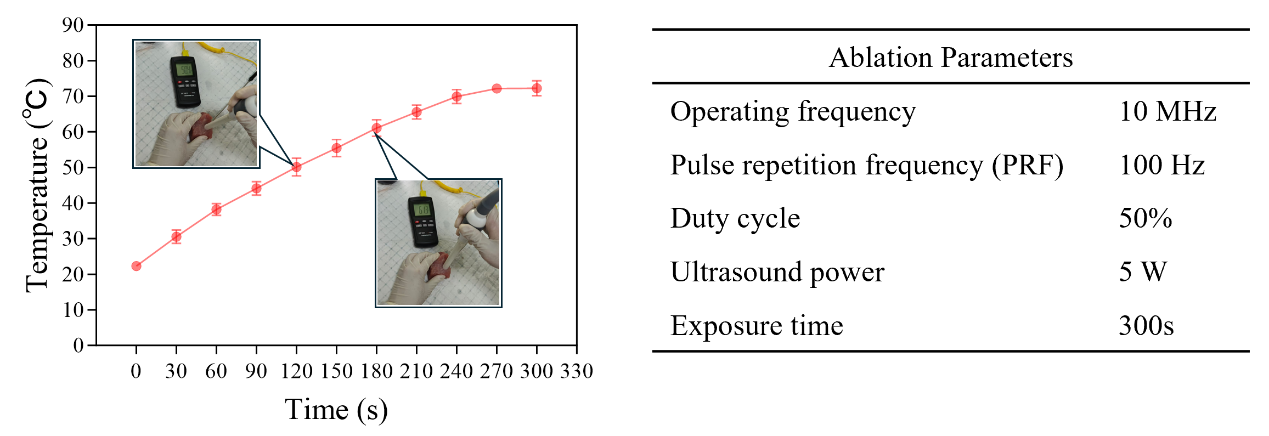


**Figure S11.** Temperature rise curve and ablation parameters. (a) Temperature change at the focal point of HIFU ablation on pork tissue over time. (b) Parameters used for rabbit thyroid ablation.

**a**

**b**


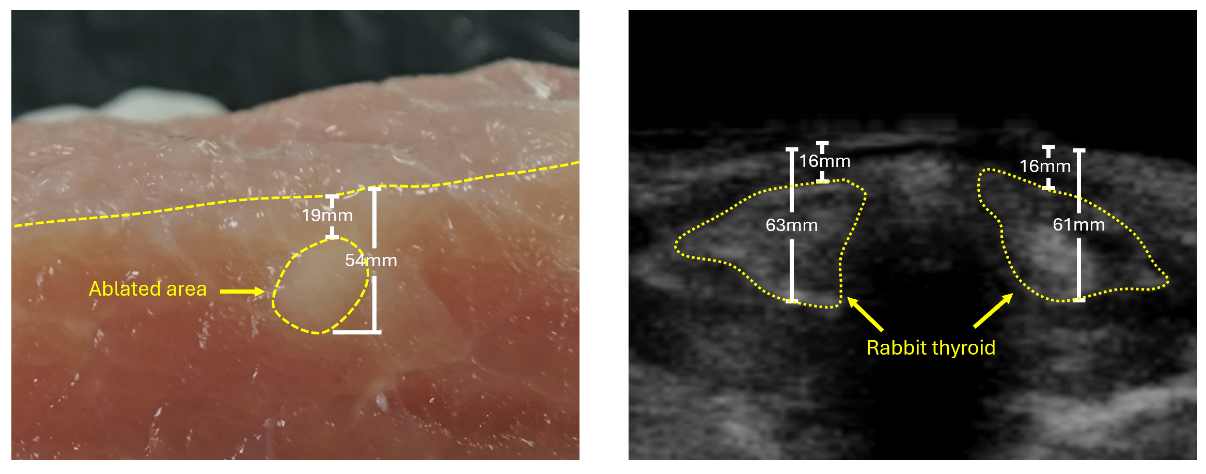


**Figure S12.** The comparison of ablation site and rabbit thyroid. (a) Pork tissue after HIFU ablation, illustrating the depth profile of ablated area. (b) Ultrasound images of the rabbit thyroid, measuring the depth profile of each thyroid.


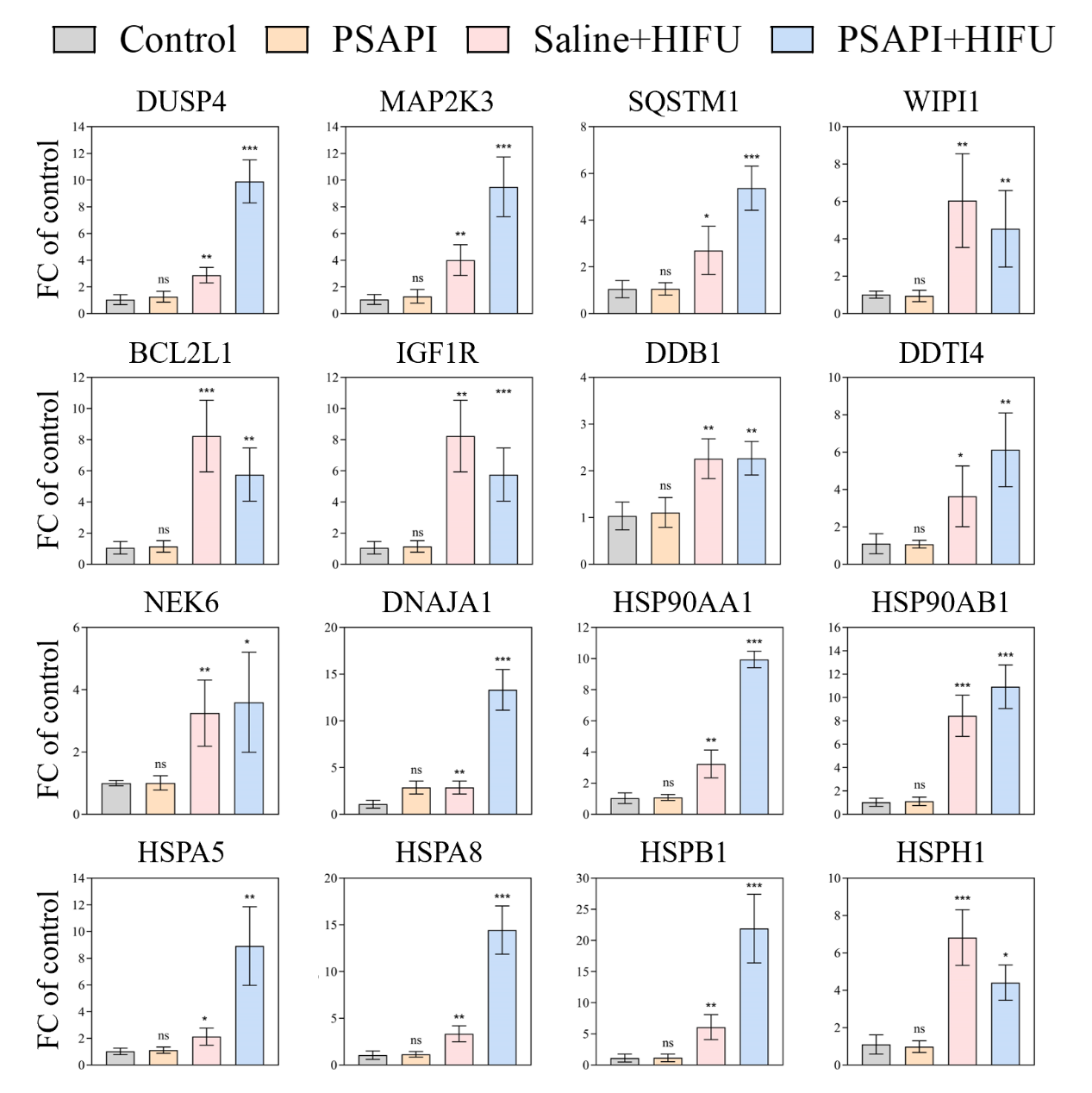


**Figure S13.** RT-qPCR validation of cell damage-repair related gene expression in mice after HIFU treatment. Data are presented as mean ± SD (n = 4). Statistical significance was determined using one-way ANOVA followed by Dunnett’s multiple comparison test. ns, not significant, *p < 0.05, **p < 0.01, and ***p < 0.001.


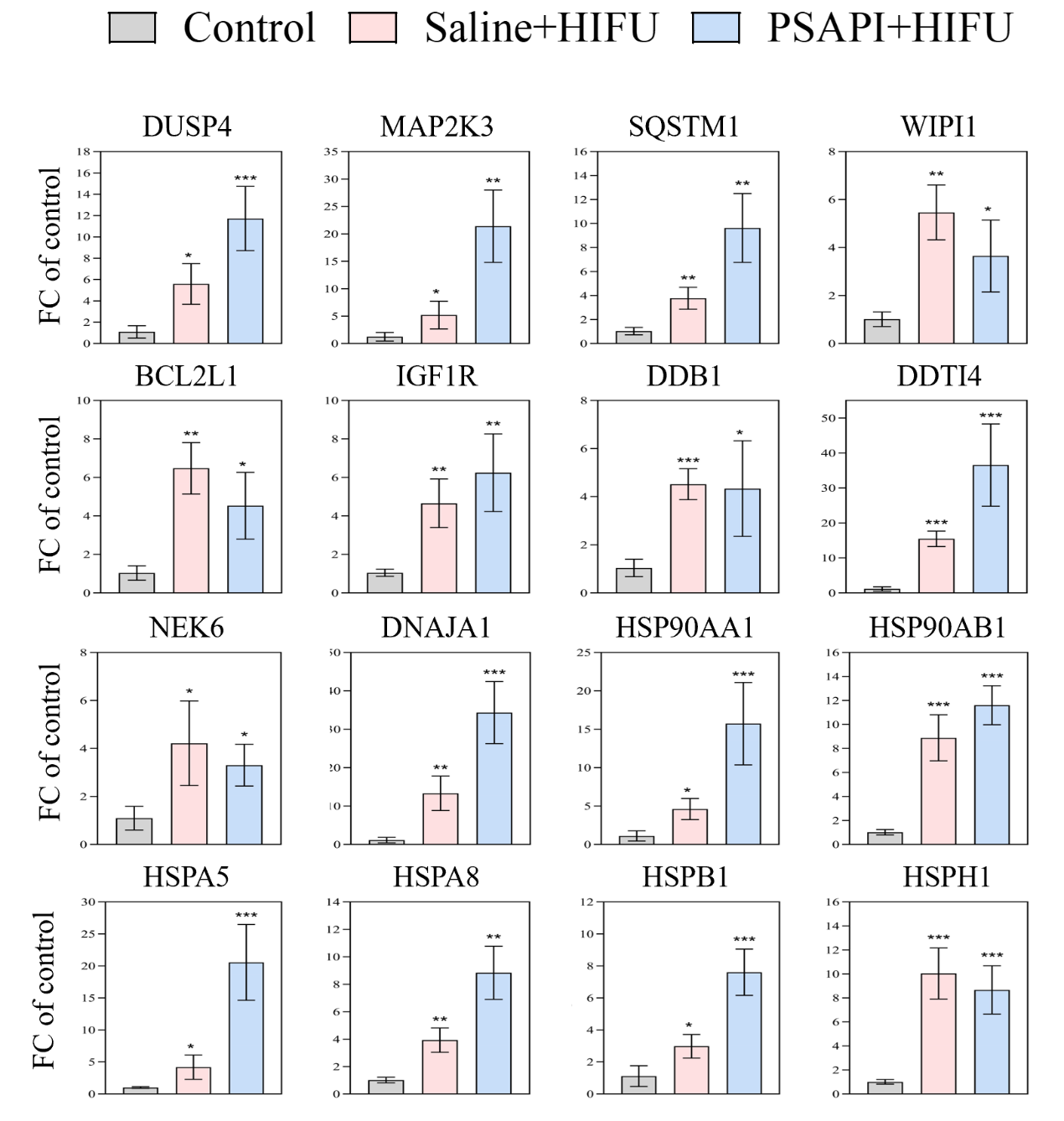


**Figure S14.** RT-qPCR validation of cell damage-repair related gene expression in rabbits after HIFU treatment. Data are presented as mean ± SD (n = 4). Statistical significance was determined using one-way ANOVA followed by Dunnett’s multiple comparison test. ns, not significant, *p < 0.05, **p <0.01, and ***p < 0.001.


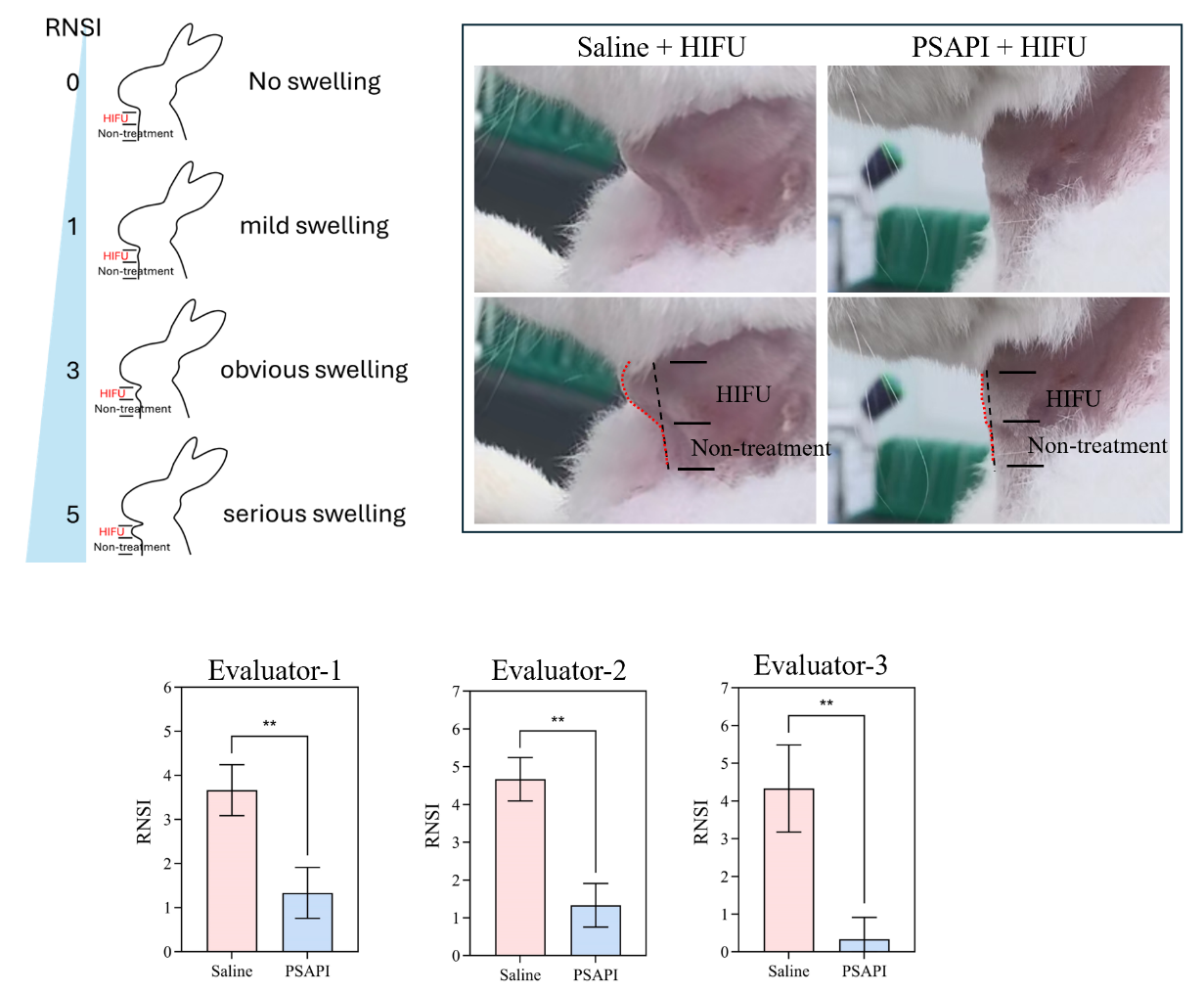


**b**

**a**

**Figure S15.** Accessing of RNSI (Rabbit neck swelling index). (a) Diagram of definition of RNSI and photos of the representative lateral view of the rabbit neck. (b) RNSI rating by different evaluators. Data are presented as mean ± SD (n = 3). Statistical significance was determined using t test. *p < 0.05 and **p < 0.01 versus Saline.


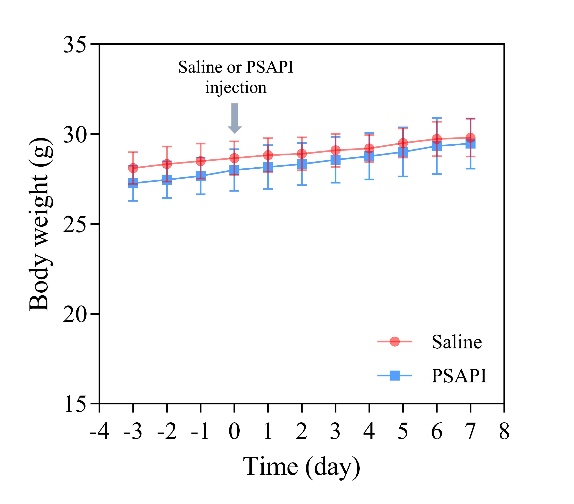


**Figure S16.** Body weight of mice before and after PSAPI injection.


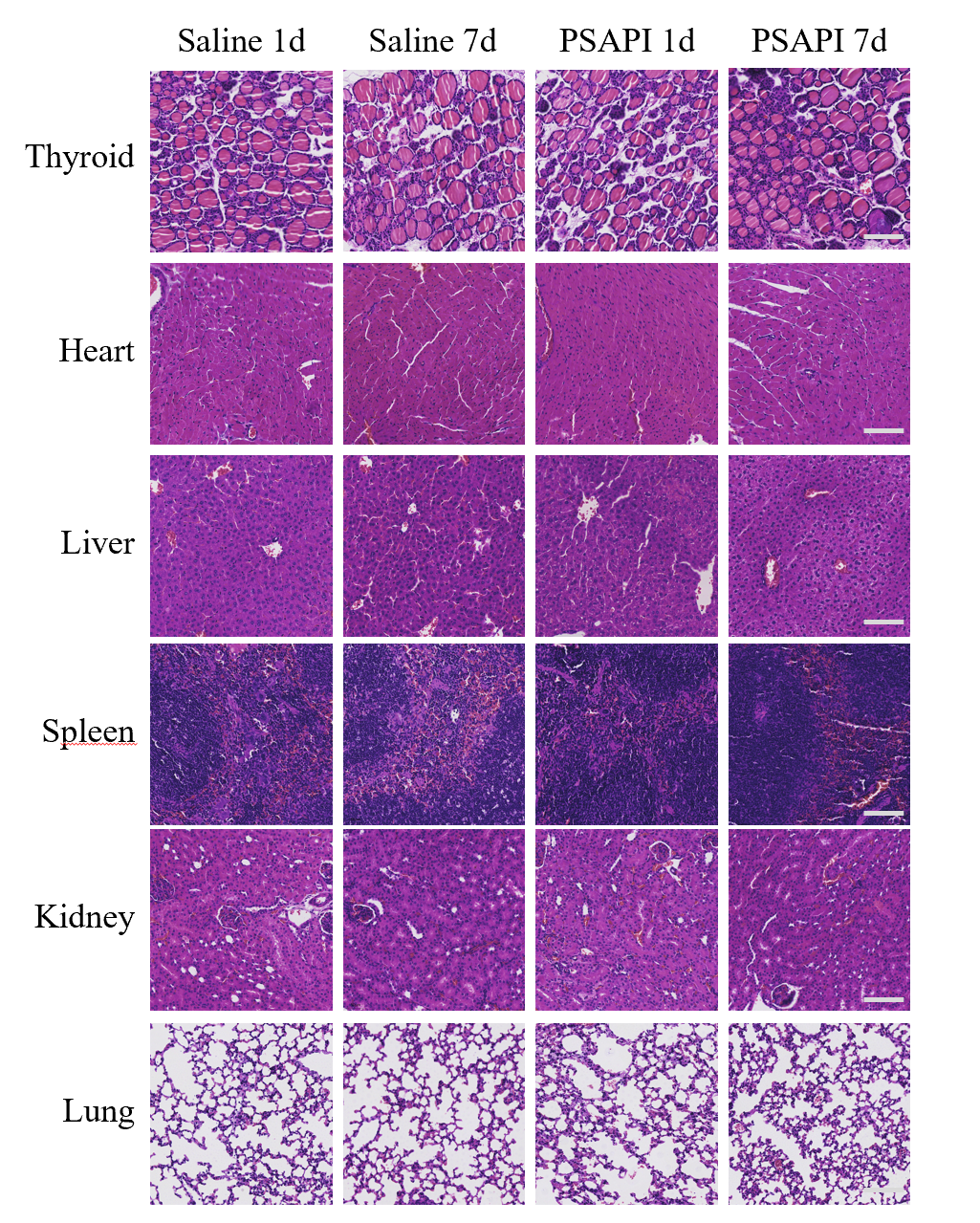


**Figure S17.** H&E staining of the major organs of mice. Scale bar: 100 μm.


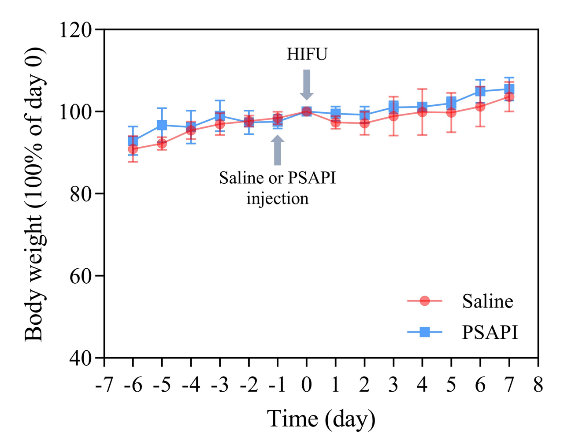


**Figure S18.** Body weight of rabbits before and after PSAPI injection and HIFU treatment.


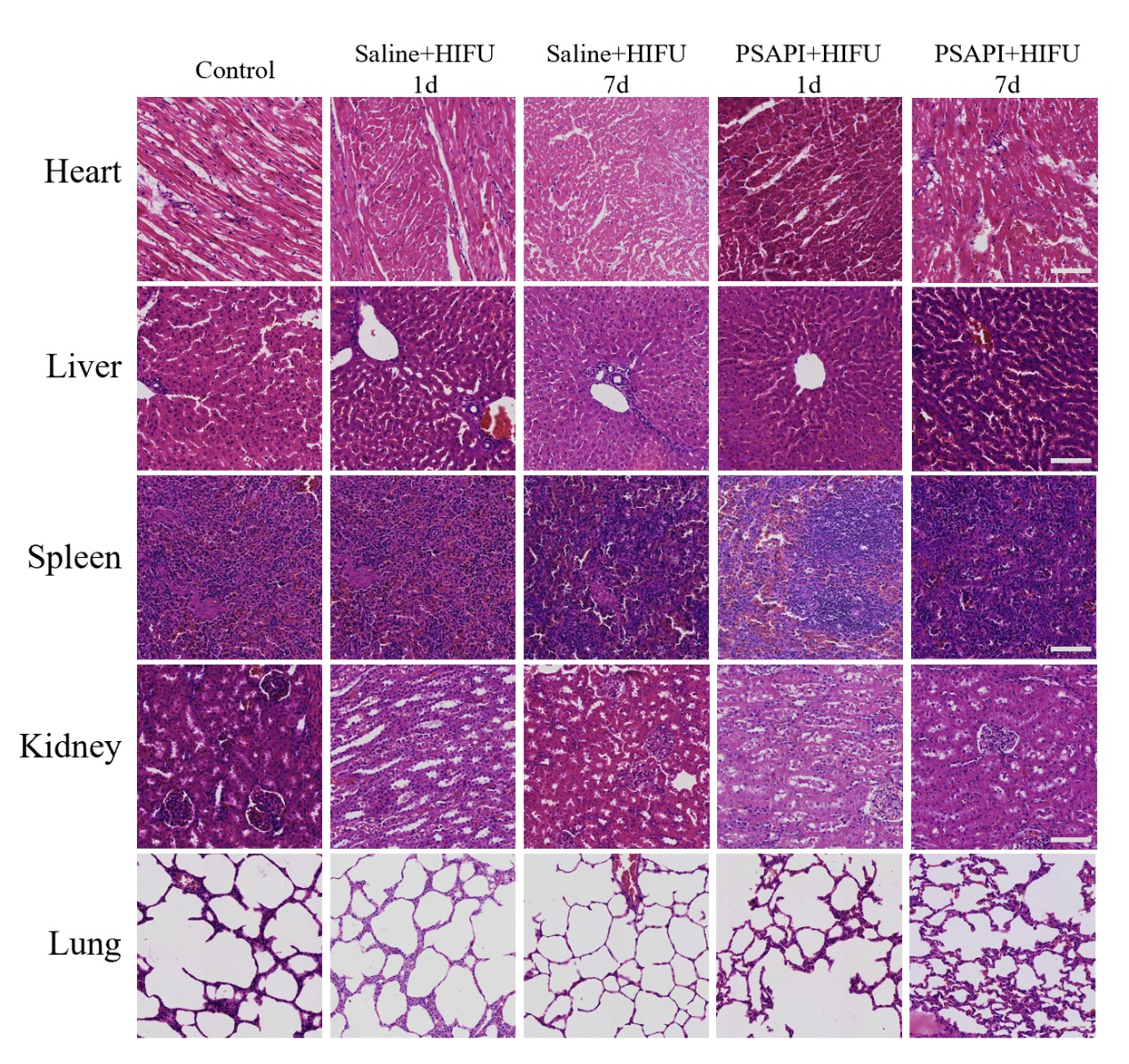


**Figure S19.** H&E staining of the major organs of rabbits. Scale bar: 100 μm.


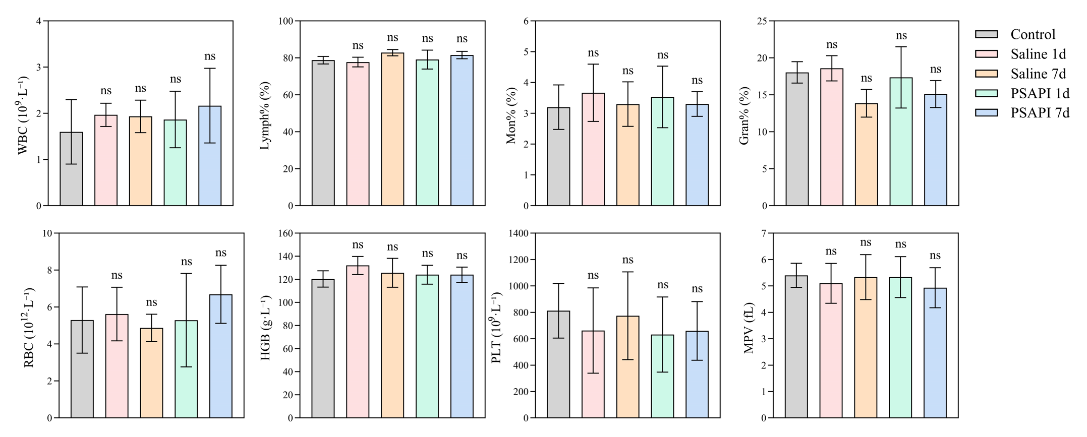


**Figure S20.** Blood routine of mice, including white blood cell count (WBC), lymphocyte percentage (Lymph%), monocyte percentage (Mon%), granulocyte percentage (Gran%), red blood cell count (RBC), hemoglobin (HGB), platelet count (PLT), and mean platelet volume (MPV). Data are presented as mean ± SD (n = 3). Statistical significance was determined using one-way ANOVA followed by Dunnett’s multiple comparison test. ns, not significant.


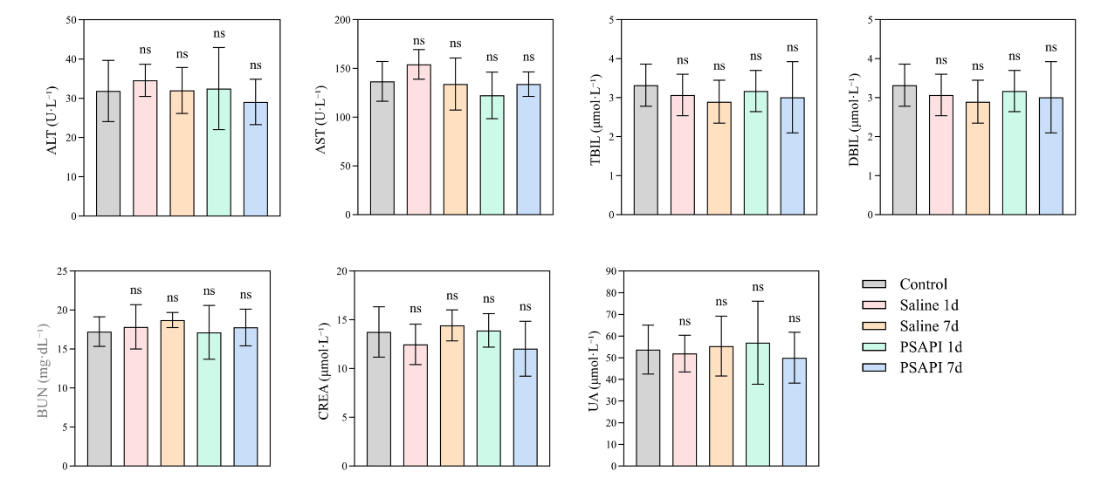


**Figure S21.** Blood biochemistry of mice, including liver function: alanine aminotransferase (ALT), glutamic oxaloacetic transaminase (AST), total bilirubin (TBIL) and direct bilirubin (DBIL); renal function: blood urea nitrogen (BUN), creatinine (CREA) and uric acid (UA). Data are presented as mean ± SD (n = 3). Statistical significance was determined using one-way ANOVA followed by Dunnett’s multiple comparison test. ns, not significant.


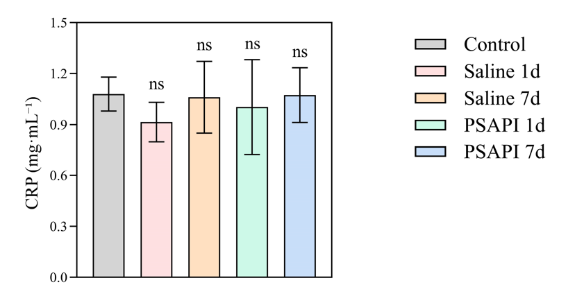


**Figure S22.** C-reactive protein (CRP) levels of mice. Data are presented as mean ± SD (n = 3). Statistical significance was determined using one-way ANOVA followed by Dunnett’s multiple comparison test. ns, not significant.

**Table 1**. List of primers of mice used in this study.

| Gene | Primer Sequences (5'-3') |
| --- | --- |
| GAPDH | F： GAAGGGCTCATGACCACAGT |
|  | R： TGCAGGGATGATGTTCTGGG |
| IL-6 | F： CCCACCAGGAACGAAAGTCA |
|  | R： ACTGGCTGGAAGTCTCTTGC |
| IL-10 | F： GCATGGCCCAGAAATCAAGG |
|  | R： ACACCTTGGTCTTGGAGCTTATTA |
| TNF-α | F： ACCCTCACACTCACAAACCAC |
|  | R： ACAAGGTACAACCCATCGGC |
| TGF-β | F： ACTGGAGTTGTACGGCAGTG |
|  | R： GGGGCTGATCCCGTTGATTT |
| DUSP4 | F： CCTCTACTCGGCTGTCATCG |
|  | R： ACCTCTCATAGCCACCTTTAAGC |
| MAP2K3 | F： GGGAGATCGCTGTGTCTATCG |
|  | R： GTCGCACATCTTCACATGCC |
| SQSTM1 | F： GGACCCATCTACAGAGGCTG |
|  | R： ATCACAATGGTGGAGGGTGC |
| WIPI1 | F： CCAGAAGAGCCTTCCACCTG |
|  | R： GGCAGTTTCTGGATCGTGGA |
| BCL2L1 | F： CACCTCATCAGTCAGGGTGAG |
|  | R： GTGAGTGGACGGTCAGTGTC |
| IGF1R | F： GCACCAATGCTTCAGTCCCT |
|  | R： GTCTTTGGAGCAGTAGTTGTGC |
| DDB1 | F： GGCCAACAATAGCACCCTCA |
|  | R： CCACTCGTGTCTTGGACTTC |
| DDIT4 | F： CCAGAGAAGAGGGCCTTGAC |
|  | R： CATCCAGGTATGAGGAGTCTTCC |
| NEK6 | F： GCCTGCTGTATGAGATGGCA |
|  | R： CAATGTCAGGTCGGTGGTCA |
| DNAJA1 | F： GGCGAGAGTGGGCTGTAAA |
|  | R： GGGTGGTACTTCAAGGCCAA |
| HSP90AA1 | F： AATTCATCGGACGCTCTGGA |
|  | R： TCCACAATGGTCAGGGTTCG |
| HSP90AB1 | F： GCTTTCCCGTCAAGATGCCT |
|  | R： AGAGATCAACTCGCGGAGGA |
| HSPA5 | F： CGTGTGTGTGAGACCAGAAC |
|  | R： CACAGTGAACTTCATCATGCCG |
| HSPA8 | F： GGAAAGACCGTTACCAACGC |
|  | R： TTCCTTTCAGCTCCGACCTTC |
| HSPB1 | F： TCACCCGGAAATACACGCTC |
|  | R： GGCCTCGAAAGTAACCGGAA |
| HSPH1 | F： CTCGGATGTGGATGCAAATGA |
|  | R： GGCTTCTACAGGCAGCTCAA |

**Table 2**. List of primers of rabbits used in this study.

| Gene | Primer Sequences (5'-3') |
| --- | --- |
| GAPDH | F： ACCATCTTCCAGGAGCGAGA |
|  | R： GGTTCACGCCCATCACAAAC |
| IL-6 | F： CGGCGGTGAATAATGAGACCT |
|  | R： CACTCCTGAACTTGGCCTGA |
| IL-10 | F： CTGCGACAATGTCACCGATT |
|  | R： ACTCTAGCCGAGTTGCCATC |
| TNF-α | F： CTCAGGAGGAAGAGTCCCCA |
|  | R： GGTTTGCTACTACGTGGGCT |
| TNF-β | F： GATGTCACGCCTGCCTAGAA |
|  | R： CCCTTCCCTGCCTCTAGTCT |
| DUSP4 | F： AGTACAAGTGCATCCCGGTG |
|  | R： GCACTCCTTCACCGCATCTAT |
| MAP2K3 | F： AGTCCAAAGGAAAATCCAAGAGG |
|  | R： CCACCTCGAAGTTCCTGTCTC |
| SQSTM1 | F： AGCTGAGACATGGGCACTTC |
|  | R： CTCTGATGGACCAGAAGCTGA |
| WIPI1 | F： CTGACCACAGGCGAGATTGT |
|  | R： CGGATGACTGTGCCCTTTTC |
| BCL2L1 | F： CACAAAGAAACGAGTTTTGAGCC |
|  | R： CCAGCTGTATCCTTTCTGCGA |
| IGF1R | F： CACCAACGCCTCAGTTCCTT |
|  | R： CTTCTTTGGAGCAGTAGTTGTGC |
| DDB1 | F： AGAGCGTGGGCAAGATTGAA |
|  | R： CACTGCCATCGTCGTACTGT |
| DDIT4 | F： CTTTGGGACCGCTTCTCGT |
|  | R： AGGTACGCGGAGTCTTCCTC |
| NEK6 | F： GAGAGGATCCACGAGAACGG |
|  | R： GCAGAGCGGCCATCTCATAC |
| DNAJA1 | F： TCAGCCCGTTCACCGTTTTCT |
|  | R： TTTCAGTTCTTCCTGGGTGGC |
| HSP90AA1 | F： AGACCCAAGACCAGCCGAT |
|  | R： ATCTTGTCCAGAGCATCCGA |
| HSP90AB1 | F： TTTCCCTTCAAGATGCCCGA |
|  | R： GGGTCCGTCAGGCTCTCATA |
| HSPA5 | F： TCGAAGAGCTGAACATGGACC |
|  | R： ATGCGAGTAGAGCCACCAAC |
| HSPA8 | F： GGTATAAGAGGCAGGGTGGC |
|  | R： CCCTTAGACATGATTGCTTGCG |
| HSPB1 | F： TGGACGTGAACCACTTCGC |
|  | R： TCTTCGTGTTTGCCCGTGA |
| HSPH1 | F： CGAGAAGGCTCGAGAAGAAGG |
|  | R： ATCCAAATGATATGAACCGGCG |

**Reference**

[1] L. Tegler, J. Gillquist, B. Anderberg, B. Lundström, H. Johansson, J. Endocrinol. Invest. 1981, 4, 335.

[2] R. Sender, S. Fuchs, R. Milo, PLOS Biol. 2016, 14, e1002533.
